# Frontotemporal dementia subtyping using machine learning, multivariate statistics and neuroimaging

**DOI:** 10.1093/braincomms/fcaf065

**Published:** 2025-02-11

**Authors:** Amelie Metz, Yashar Zeighami, Simon Ducharme, Sylvia Villeneuve, Mahsa Dadar

**Affiliations:** Douglas Research Center, Montreal, Canada H4H 1R3; Department of Psychiatry, McGill University, Montreal, Canada H3A 1A1; Douglas Research Center, Montreal, Canada H4H 1R3; Department of Psychiatry, McGill University, Montreal, Canada H3A 1A1; Douglas Research Center, Montreal, Canada H4H 1R3; McConnell Brain Imaging Centre, Montreal Neurological Institute, McGill University, Montreal, Canada H3A 2B4; Douglas Research Center, Montreal, Canada H4H 1R3; Department of Psychiatry, McGill University, Montreal, Canada H3A 1A1; Douglas Research Center, Montreal, Canada H4H 1R3; Department of Psychiatry, McGill University, Montreal, Canada H3A 1A1

**Keywords:** magnetic resonance imaging, machine learning, frontotemporal dementia, classification, neurodegeneration

## Abstract

Frontotemporal dementia (FTD) is a prevalent form of early-onset dementia characterized by progressive neurodegeneration and encompasses a group of heterogeneous disorders. Due to overlapping symptoms, diagnosis of FTD and its subtypes still poses a challenge. Magnetic resonance imaging (MRI) is commonly used to support the diagnosis of FTD. Using machine learning and multivariate statistics, we tested whether brain atrophy patterns are associated with severity of cognitive impairment, whether this relationship differs between the phenotypic subtypes and whether we could use these brain patterns to classify patients according to their FTD variant. A total of 136 patients (70 behavioural variant FTD, 36 semantic variant primary progressive aphasia and 30 non-fluent variant primary progressive aphasia) from the frontotemporal lobar degeneration neuroimaging initiative (FTLDNI) database underwent brain MRI and clinical and neuropsychological examination. Deformation-based morphometry, which offers increased sensitivity to subtle local differences in structural image contrasts, was used to estimate regional cortical and subcortical atrophy. Atlas-based associations between atrophy values and performance across different cognitive tests were assessed using partial least squares. We then applied linear regression models to discern the group differences regarding the relationship between atrophy and cognitive decline in the three FTD phenotypes. Lastly, we assessed whether the combination of atrophy and cognition patterns in the latent variables identified in the partial least squares analysis could be used as features in a machine learning model to predict FTD subtypes in patients. Results revealed four significant latent variables that combined accounted for 86% of the shared covariance between cognitive and brain atrophy measures. Partial least squares-based atrophy and cognitive patterns predicted the FTD phenotypes with a cross-validated accuracy of 89.12%, with high specificity (91.46–97.15%) and sensitivity (84.19–93.56%). When using only MRI measures and two behavioural tests in the partial least squares and classification algorithms, ensuring clinical feasibility, our model was equally precise in the same participant sample (87.18%, specificity 76.14–92.00%, sensitivity 86.93–98.26%). Here, including only atrophy or behaviour patterns in the analysis led to prediction accuracies of 69.76% and 76.54%, respectively, highlighting the increased value of combining MRI and clinical measures in subtype classification. We demonstrate that the combination of brain atrophy and clinical characteristics and multivariate statistical methods can serve as a biomarker for disease phenotyping in FTD, whereby the inclusion of deformation-based morphometry measures adds to the classification accuracy in the absence of extensive clinical testing.

## Teaser

Metz *et al*. report that machine learning can effectively differentiate frontotemporal dementia subtypes, combining brain atrophy patterns and cognitive performance. Using deformation-based morphometry and multivariate statistics, they achieved subtype classification with high accuracy, sensitivity and specificity. This approach demonstrates potential as a biomarker for phenotyping frontotemporal dementia in clinical settings.

## Graphical Abstract

**Graphical Abstract:**
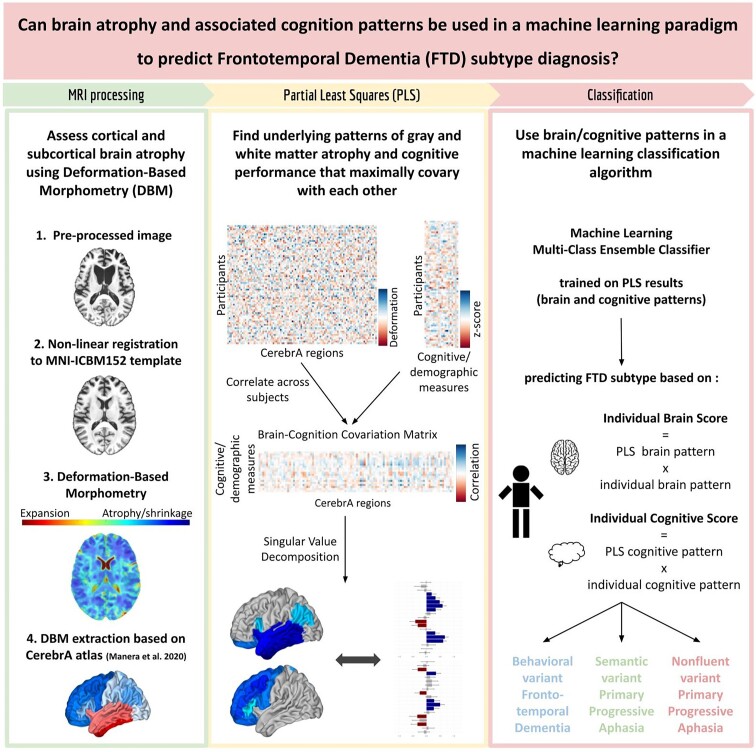

## Introduction

Frontotemporal dementia (FTD) is one of the most common forms of early-onset dementia. It is characterized by atrophy and gliosis in the frontal and temporal lobes of the brain^1^ as well as neuropathological abnormalities in the form of hyperphosphorylated protein accumulations, typically composed of either tau or TDP-43.^2,3^ Clinically, it encompasses a group of heterogeneous neuropathological disorders causing a wide spectrum of symptoms, including changes in behaviour, language, executive control and motor symptoms.^4,5^ The core FTD spectrum syndromes are behavioural variant FTD (bvFTD), non-fluent variant primary progressive aphasia (nfvPPA) and semantic variant primary progressive aphasia (svPPA). FTD also includes subtypes that primarily affect movement, namely progressive supranuclear palsy and corticobasal syndrome. Patients with bvFTD initially present with abnormal behaviour, changes in personality and emotion and reduced executive control and social cognition. This includes symptoms like disinhibition, compulsions, dietary changes, apathy or lack of empathy.^6^ In primary progressive aphasias, cognitive deficits predominantly manifest themselves in the language domain. About 5–7 years after symptom onset, as pathology spreads,^5^ patients develop additional behavioural symptoms of bvFTD. Patients with svPPA show progressive impairments in conceptual knowledge, word retrieval and single-word comprehension whereas nfvPPA is defined by effortful speech in combination with motor speech apraxia and agrammatism.^7^

The diagnosis of FTD and its subtypes still poses a significant challenge to clinicians.^8^ Recent research highlights that FTD subtypes do not fall into clear, mutually exclusive categories based on clinical symptoms or structural brain alterations. Instead, patients frequently exhibit diagnostic characteristics associated with multiple subtypes, as impairments in behaviour, movement and language often overlap across diagnostic boundaries. This lack of distinct separation between subtypes complicates the diagnostic process, as patients may simultaneously present features that fit various FTD-related disorders.^9,10^ Structural magnetic resonance imaging (MRI) is commonly used in clinical practice to confirm a diagnosis of FTD.^11^ However, classical MRI metrics are currently insufficiently sensitive to detect subtle neuronal loss in the early stages of the disease,^12^ leading to delayed or incorrect diagnoses. The development of biomarkers for the detection and diagnosis of FTD at symptom onset is critical to ensure optimal care for patients as well as to accurately inform critical trials. As methods of morphometric analysis and the use of multivariate statistics and machine learning methods are advancing, it is becoming increasingly feasible to combine MRI-based features with these techniques to improve early detection and diagnosis.

Previous MRI studies on FTD have largely focused on assessing grey matter atrophy as measured by cortical thickness or voxel-based morphometry.^11^ The findings of these studies corresponded to evidence from post-mortem studies in that they demonstrated specific patterns of atrophy in frontal and temporal cortices on a group level.^13,14^ bvFTD has been related to atrophy in fronto-insular cortices as well as basal ganglia.^15-18^ svPPA has been associated with left anterior temporal pole and hippocampal atrophy.^19-22^ In nfvPPA, atrophy seems to be most prevalent in the left inferior frontal gyrus, specifically involving Broca’s area, and will include motor regions if comorbid with speech apraxia.^13,23-26^ More recent results indicate that despite interindividual differences in atrophy maps, regions affected by atrophy tend to be functionally connected to the brain regions typically associated with the respective FTD variant as well as to symptom-specific networks.^14^ Another method to estimate atrophy patterns in MRI images is deformation-based morphometry (DBM; see ‘Materials and methods’ section for a description of this technique). Using DBM offers significant advantages for estimating cortical atrophy. Unlike voxel-based morphometry and cortical thickness estimation, which rely on automated tissue segmentation and risk incorrect grey matter volume calculations due to misclassifications,^27^ DBM leverages image contrast directly to capture tissue changes without segmentation. This method is also more sensitive to subtle differences because it does not require smoothing and matches images locally (i.e. at the voxel level), enabling the detection of partial changes within larger volumes.^28,29^ Additionally, DBM measures changes in both white matter and subcortical grey matter, unlike cortical thickness estimation.^30^ This makes DBM a potential candidate for diagnostic purposes, given the need for improved diagnostic biomarkers for FTD and particularly for the categorization of patients according to FTD variants. However, only few studies have applied DBM in the context of FTD, generally supporting previous findings.^31,32^ Notably, Cardenas *et al*.^33^ found atrophy in FTD not only in the frontal and anterior temporal lobes but also in the thalamus, pons and superior and inferior colliculi. Moreover, Manera *et al*.^34^ emphasize changes in white matter and subcortical structures like thalamus, amygdala and basal ganglia in bvFTD and point out ventricular expansion as a common feature in this cohort. While these findings broadly align with the conclusions of other MRI studies, they also highlight the utility of DBM in identifying structural changes, especially in subcortical areas.^30^

Previous attempts at an automated classification of FTD patients based on structural MRI in combination with machine learning techniques have achieved high accuracy (80–90%) in distinguishing patients from control groups.^11^ However, few cases have implemented a multiclass approach.^35-41^ As binary classifiers necessitate the exclusion of all but two potential clinical labels, multiclass methods offer greater value from a clinical standpoint. Additionally, FTD subtypes represent a spectrum, making it challenging for a binary variable to fully encompass their nuances. Thus, we opted for a multiclass method here. Furthermore, many studies grouped FTD clinical variants together in their analysis. Considering their heterogeneity in terms of behavioural and neurodegenerative features, early detection of specific FTD syndromes is highly relevant to determine appropriate treatments. While studies achieved high accuracies,^23,42^ only two studies classified each FTD subtype against a group of all others and patients with Alzheimer’s disease. Notably, Tahmasian *et al*.^43^ achieved high specificity (97.5% and 94.2%) but very poor sensitivity (50% and 0%) in differentiating one FTD subtype from the others. Kim *et al*.^44^ used cortical thickness measures in a hierarchical classification scheme to classify FTD subtypes, which resulted in an accuracy of 75.8%. Improved implementation of machine learning is therefore crucial for enhancing early detection of FTD variants.

The present study took advantage of the sensitivity of DBM measurements to capture the relationship between brain atrophy patterns and disease-related clinical measures in the three main variants of FTD. Modelling the relationship between brain atrophy and cognitive decline individually in three phenotypic variants (bvFTD, svPPA and nfvPPA) might make it possible to disambiguate the different domains of disease within the FTD population and their link with brain morphometric measures. Here, we used a multivariate method to relate the different cognitive symptoms of FTD variants to system-wide atrophy patterns. We analysed data from 136 FTD patients who were diagnosed with either bvFTD, svPPA or nfvPPA from the frontotemporal lobar degeneration neuroimaging initiative (FTLDNI) database. We used DBM as a measure of structural brain alterations, in combination with partial least squares (PLS)^45-47^ to quantify the magnitude and pattern of volume change in different FTD variants as compared with each other and to identify the cortical and subcortical structures most sensitive to change. Using the resulting patterns that maximally explain the covariance between FTD subtypes, we were able to predict FTD variant diagnosis in our cohort.

In concordance with previous neuroimaging and *ex vivo* studies, we expected neurodegenerative changes in fronto- and temporocortical regions as well as subcortical structures. The main aim of this study was to enhance the understanding of the disease mechanisms underlying FTD and to provide potential biomarkers, composed of imaging, clinical and demographic data, for disease severity assessment and phenotyping in the absence of extensive clinical testing.

## Materials and methods

### Participants

Data analysed in this study include participants from the FTLDNI that had T1-weighted MR images. The FTLDNI was founded through the National Institute of Aging and started in 2010 (https://memory.ucsf.edu/research/studies/nifd). The primary goals of FTLDNI are to identify neuroimaging modalities and methods of analysis for tracking frontotemporal lobar degeneration and to assess the value of imaging versus other biomarkers in diagnostic roles. Baseline and follow-up data from 136 FTLDNI participants were included in this study. Data were accessed and downloaded through the LONI platform in July 2023. We included patients with bvFTD [*n*(baseline) = 70, *n*(follow-up) = 38], svPPA [*n*(baseline) = 36, *n*(follow-up) = 24] and nfPPA [*n*(baseline) = 30, *n*(follow-up) = 15]. The inclusion criteria for FTD patients were diagnosis of possible or probable FTD according to the FTD consortium criteria. For up-to-date information on participation and protocol, please visit study website. Diagnoses remained stable between baseline and follow-up visits for all participants. All participants provided informed consent, and the protocol was approved by the institutional review boards at all sites.

### Clinical assessment

All participants were assessed at the initial visit for clinical characteristics (motor, non-motor and neuropsychological performance) by site investigators. The neuropsychological assessment included Mini-Mental State Examination (MMSE) and Clinical Dementia Rating (CDR) scale as measures of global cognition, forward digit span and California Verbal Learning Test (items recalled correctly after four learning trials, items recalled correctly after 30 s delay, items recalled correctly after 10-min delay, and word recognition) as measures of verbal memory and learning, Modified Trail Making Test (time and correct lines) and backward digit span as measures of executive function, as well as verbal fluency (phonological and semantic), Boston Naming Test (BNT) and Peabody Picture Vocabulary Test as measures of language ability.

### Structural MRI acquisition and processing

The FTLDNI uses the infrastructure established by the Alzheimer’s Disease Neuroimaging Initiative. All participating imaging centres share a common platform. Available information on acquisition parameters and scanners is summarized in Supplementary Table 1. For further details on MRI acquisition protocols and scanner information, please refer to https://cind.ucsf.edu/research/grants/frontotemporal-lobar-degeneration-neuroimaging-initiative-0.

T1-weighted scans for each participant were pre-processed through our standard pipeline including noise reduction,^48^ intensity inhomogeneity correction^49^ and intensity normalization into the range (0–100). The pre-processed images were then linearly (nine parameters: three translation, three rotation and three scaling)^50^ and non-linearly^51^ registered to the MNI-ICBM152-2009c average.^52^ The quality of the linear registrations was visually verified by an experienced rater (author M.D.), blinded to the diagnostic group. Only seven scans did not pass this quality control step and were discarded.

### DBM values

DBM analysis was performed using MNI MINC tools.^34^ The principle of DBM is to warp each individual scan to a common template through a non-linear deformation, where local shape differences between the two images (i.e. the participant’s T1-weighted image and the template) are encoded in the deformations. The local deformation obtained from the non-linear transformations can then be used as a measure of tissue expansion or atrophy by estimating the determinant of the Jacobian for each transform. Local contractions can be interpreted as shrinkage of tissue (atrophy) and local expansions are often related to ventricular or sulci enlargement. DBM was used to assess regional volumetric differences whereby DBM values were calculated based on 102 regions from CerebrA atlas.^53^

### Statistical analysis

#### Demographics and cognitive scores

All statistical analyses were performed using MATLAB version R2022a. Graphs were created using R (v2024-08-01). One-way ANOVAs were conducted to compare demographic and cognitive variables at baseline, followed by independent *t*-tests with Tukey honestly significant difference correction for multiple comparisons. Categorical variables (i.e. sex) were analysed using *χ*^2^ analyses. Results are expressed as mean ± standard deviation and (median). A *P*-value of <0.05 was considered statistically significant.

#### PLS regression

PLS analysis was used to relate cognitive and brain atrophy patterns.^45,47^ The goal of the analysis is to identify combinations of cognitive scores and atrophy patterns that optimally covary with each other. PLS is a multivariate technique used to establish relationships between two sets of variables. This approach can be used to identify weighted linear combinations of variables that exhibit a high degree of covariation.^46^ The resulting linear combinations of these variables can be construed as atrophy networks and their corresponding clinical manifestations.

**Figure 1:**
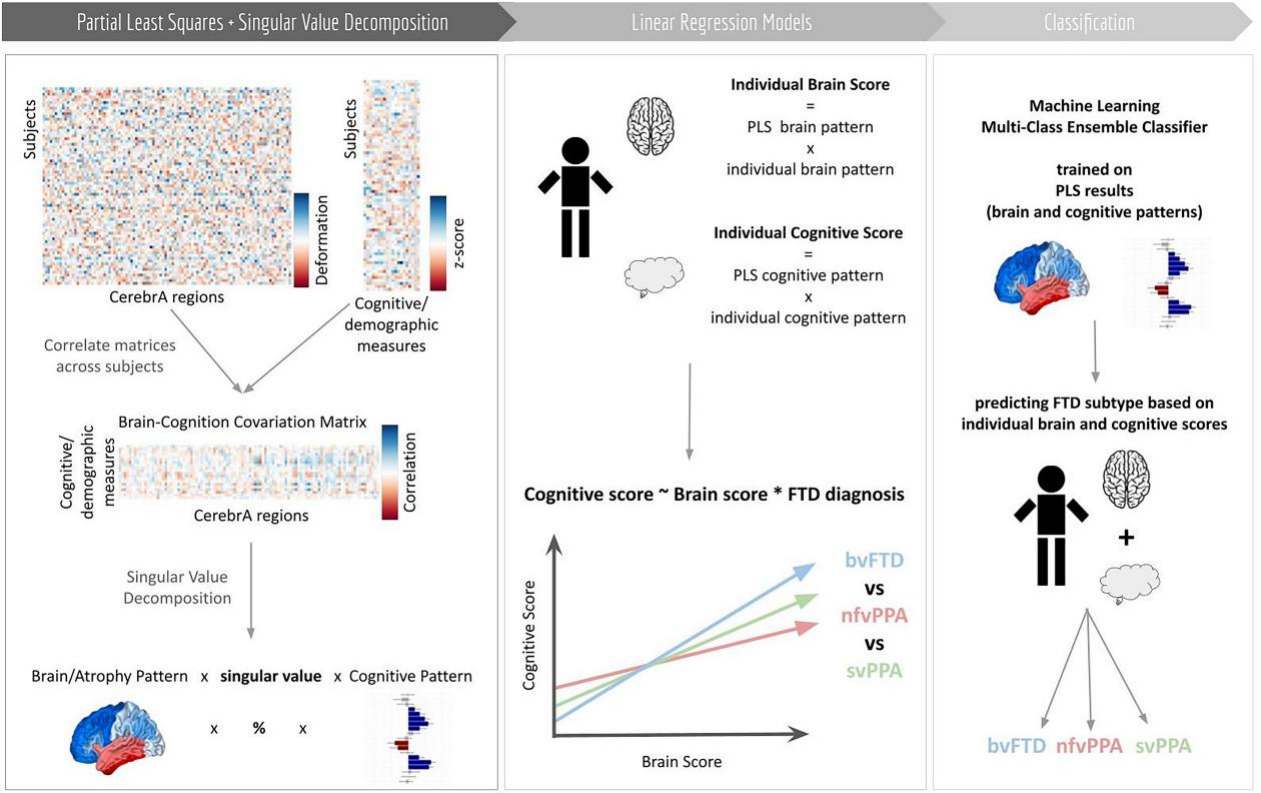
**PLS analysis flowchart.** *Z*-scored matrices for DBM data and cognitive/demographic data were combined into a single brain * cognition covariation matrix. Consequently, we applied single-value decomposition to the resulting matrix, yielding orthogonal LVs. Each LV represented atrophy patterns linked to clinical characteristics, with their associated singular value reflecting the covariance between atrophy and cognition (for detailed explanation, see Zeighami *et al*.^54^). We then applied linear regression models to discern the differences between the diagnostic FTD groups in terms of cognition and atrophy profiles in each LV. Lastly, we used the individual brain and cognition scores of each patient as features in a machine learning classifier (employing an ensemble of discriminant learners with bagging aggregation method) to predict the FTD subtype of each participant both in the baseline and follow-up visit data. bvFTD, behavioural variant FTD; FTD, frontotemporal dementia; PLS, partial least square; nfvPPA, non-fluent variant PPA; svPPA, semantic variant PP.

We followed the approach described in Zeighami *et al*.^54^ (Fig. 1). Cognitive and DBM-based data were represented as two matrices X and Y, with X representing cognitive scores in 12 columns, while Y representing brain measure across 102 regions using the CerebrA atlas. The matrices were standardized through *z*-scoring and a correlation matrix (**XʹY**) was computed. The correlation matrix was then subjected to singular value decomposition (SVD^55^).


(1) $$X^{\prime }Y=U\Delta V^{\prime }.$$

The decomposition process results in a collection of orthogonal latent variables (LVs), with U and V constituting the matrices of left and right singular vectors, while Δ is a diagonal matrix containing the singular values. The covariance explained by each LV is used as its effect size.

The statistical significance of each LV was evaluated using permutation tests. The rows of matrix X were randomly permuted (repeated 500 times), and the cognition–brain correlation matrix was recomputed. The permuted correlation matrices were subjected to singular value decomposition as before, generating the null distribution for the covariance explained across LVs, which was then used to calculate the *P*-value.

The contribution of individual variables was assessed through bootstrap resampling (repeated 500 times). As a result, a sampling distribution was generated for each individual weight within the singular vectors. A ‘bootstrap ratio’ was computed for each CerebrA region, representing the ratio of its singular vector weight to its standard error estimated through bootstrapping and used to identify regions that make substantial contributions to the atrophy patterns.^56^ Bootstrap ratio maps were thresholded using the 95% confidence interval criterion.

To ensure that age, sex or education were not driving the observed relationship between brain characteristics and cognitive function in the patients, we repeated the analyses after regressing out the effects of age, sex and education based on the healthy control group (for demographic and clinical characteristics of the control group, see Supplementary Table 2). To this end, we transformed all variables to *w*-scores.^57^ Using linear regression models including age, sex and education as covariates, we adjusted the raw patient scores relative to the mean and standard deviation of the control group. This provides a demographic-corrected score that enables more accurate assessments of individual atrophy and cognitive characteristics.

#### Group differences between FTD variants

To gauge the extent to which the data-driven PLS patterns differ between the FTD variants, patient-specific scores were computed. Specifically, the brain and cognitive profiles for LVs were projected onto individual patients’ data, producing scalar atrophy scores and cognitive scores for each participant. These scores are akin to principal component scores or factor scores:


(2) Brainscore=XU,

(3) Cognitivescore=YV.

We then used the following linear regression model to assess if the relationship between atrophy scores and cognitive scores differs between the three phenotypic FTD groups:


(4) Cognitivescore∼brainscore+variant+brainscore:variant.

The variable of interest was the interaction term, brain score:variant, which indicates the slope differences between the three FTD subtypes and reflects the contribution of cortical atrophy to cognitive performance in each diagnostic group.

#### Predictive capacity of the model

##### Classification analysis

We assessed whether the combination of atrophy and cognitive patterns identified in the PLS analysis could be used as features to predict FTD subtypes in patients. To this end, we employed a machine learning multi-class ensemble classifier, using an ensemble of discriminant learners (bagging aggregation method). We used the fitcensemble function in MATLAB version R2022a to create an ensemble classifier, combining multiple ‘learners’ to enhance predictive performance over a single model. By training multiple weak classifiers and aggregating their predictions, the ensemble yields a more robust classifier. We applied bootstrap aggregation, dividing the training data into multiple subsets via sampling with replacement. Each subset trains an individual weak learner, and the class with the majority vote across models becomes the final prediction. This approach reduces prediction variance and mitigates overfitting. We specified discriminant learners, which use Gaussian distributions to model each class (see codes available on GitHub: https://github.com/ameliemetz/FTD_subtype_prediction).

A 10-fold cross-validation scheme was performed on 100 randomized train and test splits to assess the performance of the classifier. We used the combination of neurodegenerative and cognitive patterns in the LVs identified in the PLS analysis as features to train the model. The model then predicted clinical diagnoses (i.e. FTD variants) in the test data. Performance of the model was evaluated in terms of prediction accuracy for each cross-validation fold (mean and standard deviation) by comparing the predicted diagnostic group with the clinician diagnosis (‘gold standard’) in the cross-validated test subset. We also examined the sensitivity and specificity of our model for each FTD subtype. Note that the FTD variant information was not included in any previous steps (i.e. the PLS analysis), to avoid leakage of information in the variant classification task.

##### Classification analysis with minimal input variables

To ensure the clinical utility of our diagnostic approach in terms of time and necessary neuropsychological test batteries, we repeated the PLS analysis and prediction while including minimal variables that can be completed in under an hour, i.e. only using T1-weighted MRI-derived DBM values for the brain patterns (5–10 min) and CDR (box score, language score and behaviour score, ∼30 min) and BNT (5–15 min) for the cognition score. As the full battery of tests included in our PLS analysis takes over 2 h to administer, a minimal assessment including CDR and BNT is more feasible and still informative for clinicians.

Due to missing data or non-collection of certain cognitive assessments at two of the FTLDNI research sites, analysis of the maximal model was limited to participants from the UCSF site. However, more participants had available scores for inclusion in the minimal model, allowing data from all three FTLDNI research sites to be included in the minimal model analysis. To evaluate the impact of site variability on the classification model’s performance, we repeated the minimal model analysis exclusively with participants from the University of California San Francisco (UCSF) site (for demographic and clinical characteristics of this subcohort, see Supplementary Table 3).

##### Validation of classification model

As our model included measures of disease severity, we also tested the accuracy of the predictions in a severity-matched sample, based on CDR scores (only including participants with CDR scores below 1.5), to ensure that the model does not solely rely on group differences in disease severity.

Furthermore, to account for the significant age differences in the FTD groups, with the nfvPPA group being on average 5–6.5 years older than the others, we assessed classification model performance in an age-matched sample of the NIFD cohort [mean ages bvFTD = 65.4 (SD = 5.67), svPPA = 65.1 (SD = 6.12), nfvPPA = 65.8 (SD = 6.50) years]. This ensured that the classification was not driven by the ages of participants.

Finally, we validated the stability of our model by projecting brain and cognition patterns of the LVs onto the longitudinal data to predict patients’ FTD subtype diagnosis. This analysis was included to verify the reliability of the performance of the classifier, i.e. whether it yields similar performances on different observations from the same individuals. This equates to using the follow-up data of our sample as an in-sample validation. It also allowed us to assess whether accurate classifications can be made even in later disease stages, where there might be more widespread atrophy and cognitive deficits. Using the same machine learning multi-class ensemble classifier as before, we utilized the baseline data as the training set and tested the performance of the model on the longitudinal data as the test set. Note that prediction for the longitudinal time points was also performed within the same cross-validation scheme, and no data from baseline visits of the same participants were used in the training folds for the longitudinal predictions. We normalized follow-up test and DBM scores based on the baseline data, by subtracting the mean of the baseline scores from the follow-up score and dividing by the standard deviation of baseline scores. This ensured that follow-up values were comparable with the baseline (cross-sectional) data set. We again measured the prediction accuracy, as well as sensitivity and specificity, of the model in identifying the diagnosed FTD variant of the patient.

#### Longitudinal changes

To determine the longitudinal change of the brain and cognition patterns resulting from the PLS analysis, we compared the scores of each patient with a baseline and follow-up visit (*n* = 32) using pairwise *t*-tests. Lastly, we calculated the yearly rate of change in cognitive and brain scores for each FTD syndrome to investigate whether the progression of clinical symptoms and atrophy patterns differs between diagnostic groups. We then used unpaired *t*-tests to compare the rates of change between the FTD variants.


(5) $$\Delta \;=\;\frac{\mathrm{Longitudinal}\;\;\mathrm{score}\;-\mathrm{baseline}\;\;\mathrm{score}}{\mathrm{Time}\;\;\mathrm{between}\;\;\mathrm{baseline}\;\;\mathrm{and}\;\;\mathrm{follow}\text{-}\mathrm{up}\;\;\mathrm{visit}\;\;\mathrm{in}\;\;\mathrm{years}}.$$

This analysis was repeated for the brain and cognitive scores of each LV separately. For longitudinal measurements, we selected the time point for each participant that was closest to a 1-year follow-up after the baseline assessment. The mean time between baseline and follow-up visits was 1.03 years (SD = 0.44) with a range of 0.40–3.57 years.

## Results

### Demographic and clinical characteristics

Table 1 compares demographic variables and cognitive test scores between bvFTD, svPPA and nfvPPA patients at baseline. Significant differences were observed in age distribution, with nfvPPA participants being on average older compared with other groups (bvFTD: *P* < 001; svPPA: *P* < 0.007). Clinical measures also showed notable distinctions (Supplementary Table 4). The following analyses only included those participants that had no missing values in the cognitive and demographic data (see Supplementary Table 5 for numbers of missing values and Supplementary Fig. 1 for a flowchart outlining the number of participants at each step of the analysis). As education level was primarily added as a covariate in our models, we imputed missing values as the mean values of the respective diagnostic group.

**Table 1:** Baseline demographic and cognitive characteristics in FTD subtypes

	bvFTD	svPPA	nfvPPA	*P*-value
Demographics				
Number of participants (total *n* = 136)	70	36	30	
Age (years)	61.47 (6.39)	63 (6.30)	68.10 (7.90)	<0.001*
Sex (M:F, %M)	47:23 (67.14%)	20:16 (55.56%)	14:16 (46.67%)	0.137
Education (years)	15.61 (2.88)	17.03 (2.88)	16.91 (3.30)	0.031*
Cognition				
Clinical Dementia Rating sum of boxes	6.3 (3.3)	4.1 (2.3)	2.5 (2.5)	<0.001*
Clinical Dementia Rating language subscale	0.7 (0.6)	1.1 (0.6)	1.3 (0.7)	<0.001*
Clinical Dementia Rating behaviour subscale	1.3 (0.8)	1.1 (0.6)	0.5 (0.5)	<0.001*
Mini-Mental Status Examination	23.6 (4.9)	24.4 (5.1)	25.5 (5.1)	0.287
California Verbal Learning Test memory	19.3 (7.7)	17.0 (6.5)	21.9 (7.7)	0.053
California Verbal Learning Test recall after 30 s delay	4.3 (2.7)	2.9 (2.5)	5.5 (2.9)	0.002*
California Verbal Learning Test recall after 10 min delay	3.2 (2.8)	1.8 (2.3)	5.3 (2.8)	<0.001*
California Verbal Learning Test recognition	7.1 (1.8)	6.4 (2.4)	8.2 (0.9)	0.002*
Digit span forward	5.6 (1.3)	6.8 (1.5)	5.0 (1.3)	<0.001*
Digit span backward	3.5 (1.4)	4.9 (1.3)	3.5 (1.4)	<0.001*
Modified Trail Making Test correct lines	9.9 (5.0)	13.3 (2.5)	12.2 (3.9)	0.001*
Modified Trail Making Test time	74.0 (40.4)	46.9 (29.5)	62.9 (39.1)	0.008*
Verbal fluency phonological	6.6 (4.5)	8.6 (4.5)	7.0 (5.0)	0.132
Verbal fluency semantic	9.4 (6.3)	8.1 (4.1)	11.6 (8.4)	0.137
Boston Naming Test	12.2 (3.1)	5.0 (3.6)	12.3 (2.9)	<0.001*
Peabody Picture Vocabulary Test	13.2 (3.3)	8.6 (4.2)	14.5 (2.0)	<0.001*

Values expressed as mean (standard deviation). Asterisks indicate significant group differences based on one-way ANOVA or *χ*^2^ analysis comparing the groups.

bvFTD, behavioural variant FTD; nfvPPA, non-fluent variant primary progressive aphasia; svPPA, semantic variant primary progressive aphasia.

### PLS analysis

**Figure 2:**
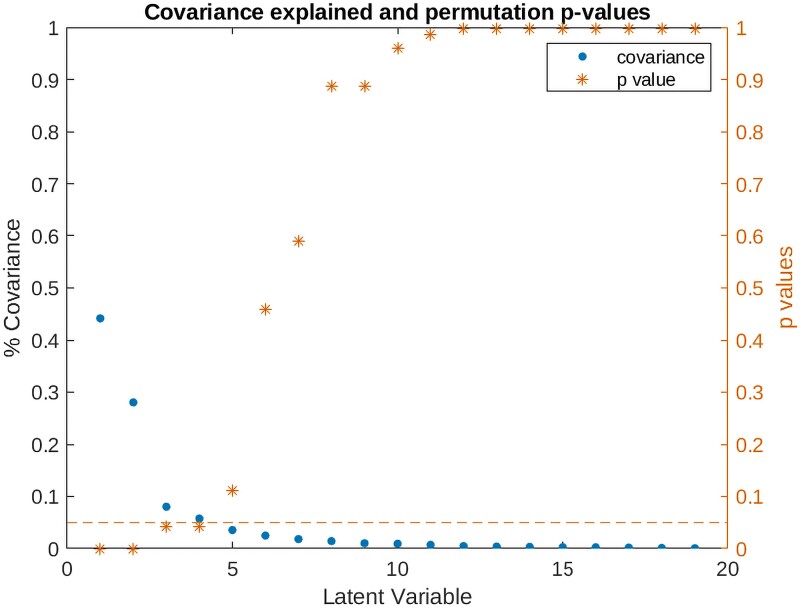
**Covariance explained and permutation *P*-values for all LVs in the PLS analysis.** PLS analysis in the maximal model including 78 FTD patients. Blue dots show the amount of covariance between cognitive and atrophy data that is explained by each LV. Orange asterisks denote *P*-values associated with each LV, based on permutation tests. LV-I (covariance explained = 44.16%, *P* < 0.001), LV-II (covariance explained = 28.05%, *P* < 0.001), LV-III (covariance explained = 8.02%, *P* < 0.05) and LV-IV (covariance explained = 5.75%, *P* < 0.05) are selected for further analysis based on *P*-value (permuted *P* < 0.05).

The PLS analysis revealed four statistically significant LVs relating clinical measures in FTD to their corresponding brain atrophy patterns (permuted *P* < 0.05) at baseline. These patterns respectively account for 44.16%, 28.05%, 8.02%, and 5.75% (total of 85.98%) of the shared covariance between clinical and brain atrophy measures (Fig. 2).

### Cognitive and atrophy patterns

**Figure 3:**
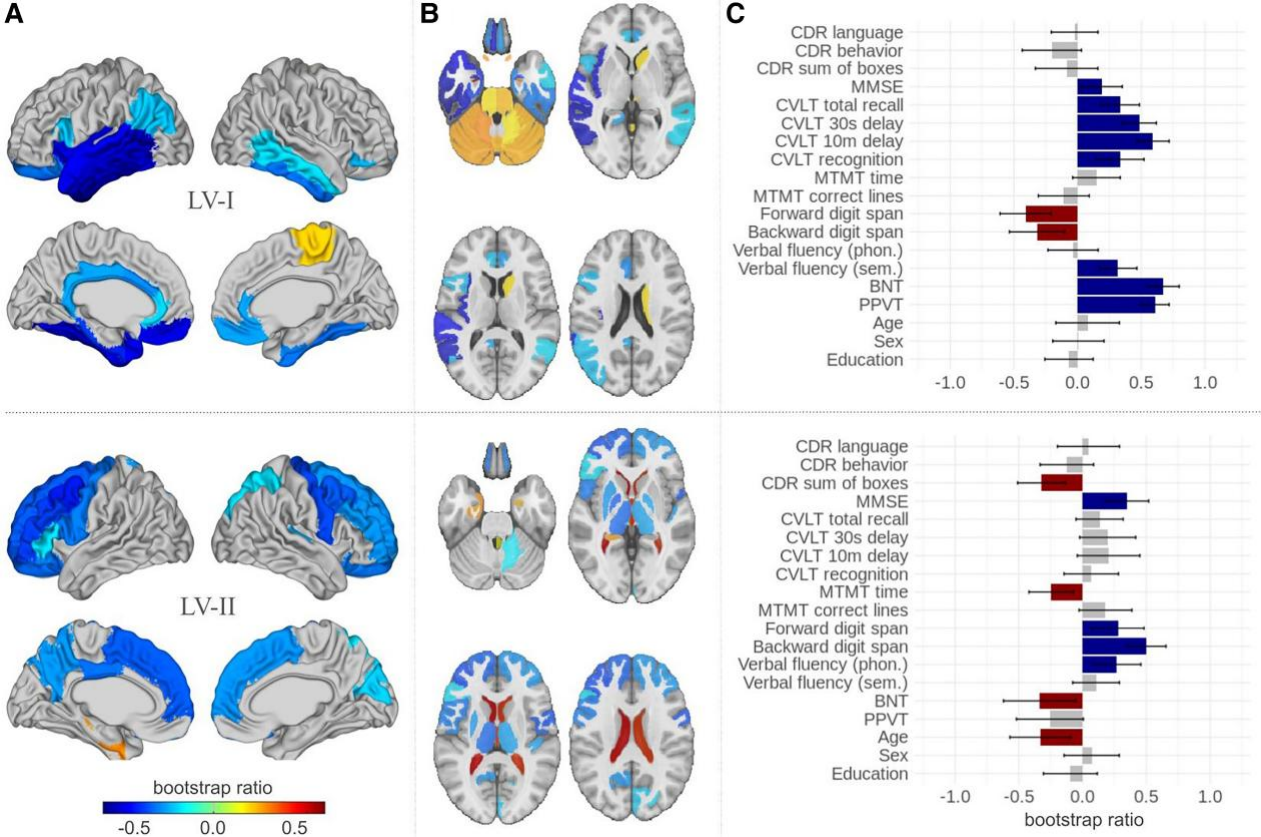
**LVs I and II obtained from the PLS analysis.** PLS analysis in the maximal model including 78 FTD patients. (**A**) Brain pattern bootstrap ratios in MNI space, surface projection. Maps only include regions that significantly contribute to the LV, as estimated by bootstrapping values (*P* < 0.05) and confidence intervals. Cooler colours denote shrinkage/atrophy, warmer colour denote expansion. (**B**) Brain pattern bootstrap values in MNI space, horizontal view illustrating subcortical structures and cerebellum. (**C**) Pattern of demographic and cognitive test scores. The effect size estimates are derived from singular value decomposition analysis and the confidence intervals, and significance levels are calculated by bootstrap resampling of the 78 participants (*P* < 0.05). Colour blue (positive values) denotes lower scores in a test, and red (negative values) denotes higher scores. BNT, Boston Naming Test; CDR, Clinical Dementia Rating; CVLT, California Verbal Learning Test; LV, latent variable; MTMT, Modified Trail Making Test; PPVT, Peabody Picture Vocabulary Test.

In summary, LV-I mostly represented confrontational naming and verbal learning/memory skills and was associated with temporal lobe structure and subcortical areas. LV-II captured diverse cognitive features in combination with frontal and subcortical atrophy patterns, while LV-III and LV-IV encompassed behavioural aspects and distributed atrophy networks (Fig. 3).

The cognitive features contributing to the first LV (LV-I), in order of magnitude, were impaired performance in confrontational naming (BNT and Peabody Picture Vocabulary Test), verbal learning (California Verbal Learning Test), semantic verbal fluency and MMSE, but comparatively higher scores in working memory/executive function (digit span). The corresponding brain pattern for this clinical profile was largely driven by atrophy in the temporal lobes and subcortical areas (amygdala, nucleus accumbens and hippocampus) on the one hand and higher volume in the cerebellum on the other hand (see Supplementary Tables 6 and 7 for specific brain areas and clinical scores).

For the second LV (LV-II), greater impairment in CDR sum of boxes, higher age and higher BNT score, as well as lower MMSE scores, digit span and phonological verbal fluency contributed to covariation. Regarding DBM measurements, predominantly increased ventricular expansion and lower volume in left frontal areas (including Broca’s area) and subcortical structures (pallidum, thalamus, but increased hippocampal volume) were involved.

Supplementary Figure 2 shows an example of how the putative brain network and the associated clinical phenotype relate to each other. For each LV, we estimated patient-specific scores by projecting the brain and cognition patterns onto individual patients’ data (see ‘Materials and methods’ section). The resulting scalar values (termed brain scores and cognition scores) reflect the extent to which an individual patient expresses each pattern. The two scores are correlated; i.e. patients with greater atrophy in the network in Fig. 3A and B also tend to conform more closely to the clinical phenotype in Fig. 3C. In LVs I and II, patients who score highly on both likely have more severe pathology, and we illustrate this by colouring the points (individual patients) by their MMSE scores. Individuals with more pronounced atrophy and symptom severity also tend to score highly on MMSE, a measure of global cognition.

Brain and cognition patterns for LVs III and IV are shown and discussed in Supplementary Fig. 3 and Supplementary Table 8.

### Group differences

**Figure 4:**
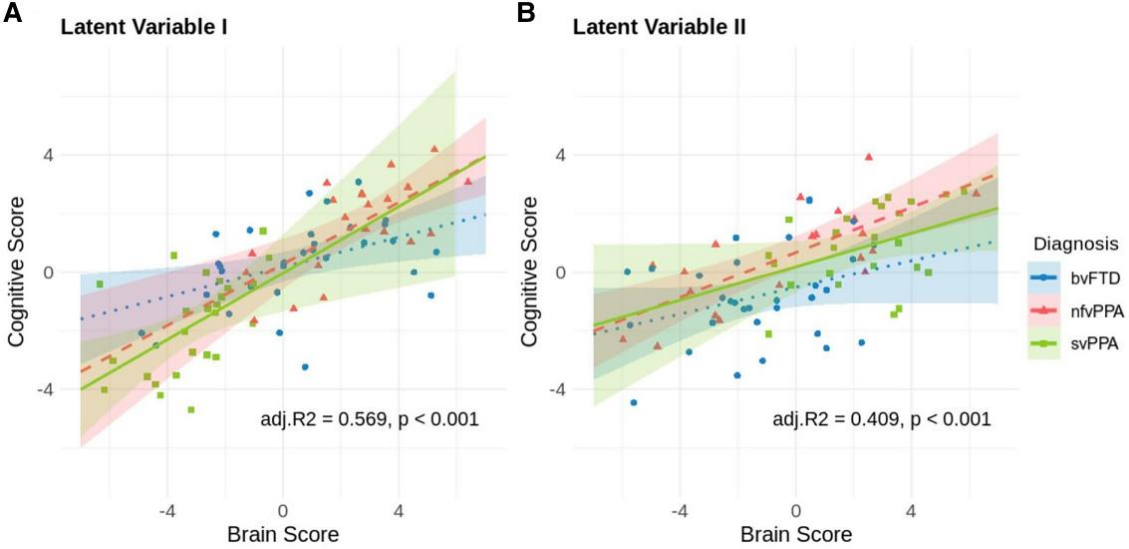
**Individual patients’ brain versus cognitive PLS score and group differences between FTD variants.** Individual brain and cognitive scores were calculated by projecting (multiplying) brain and cognitive profiles for each LV onto each patient’s data. We then applied linear regression models to compare the association of brain and cognitive scores between FTD subtypes. Each data point represents one patient (*N* = 78). (**A**) LV-I (adjusted *R*^2^ = 0.569, *P* < 0.001). (**B**) LV-II (adjusted *R*^2^ = 0.409, *P* < 0.001). bvFTD, behavioural variant FTD (dotted line); nfvPPA, non-fluent variant primary progressive aphasia (dashed line); svPPA, semantic variant primary progressive aphasia (solid line).

Figure 4 shows how the putative atrophy network and associated cognitive profiles are related to each other in individual patients and how these associations differ between FTD variants. While this measure is derived from the overall population, when compared between the groups of interest, it can provide further insight regarding the heterogeneity and nuances of the disease subgroups as it indicates whether the relationship and the degree of pattern expression are consistent or diverse across groups.

Compared with bvFTD, the brain and cognition scores in LV-I had stronger associations (i.e. steeper slopes) in svPPA and nfvPPA (Fig. 4A), although these slope differences were statistically marginal (*P* ∼ 0.08; see Table 2). For LV-II, the nfvPPA subtype had a significantly higher intercept (*tStat* = 2.57, *P* = 0.01) and slope (*tStat* = 2.19, *P* = 0.03) compared with the bvFTD variant, suggesting that it expresses the cognitive pattern to a greater extent and that the atrophy pattern has a more pronounced impact on cognition patterns in patients with nfvPPA; i.e. LV-II is reflective of the cognition and atrophy patterns of nfvPPA.

**Table 2:** Differences in the predictions of the PLS model for the different FTD subtypes in LVs I and II, as determined by a linear regression model

Contrast		*tStat*	*P*-value
Latent variable I			
bvFTD versus svPPA	Intercept	0.305	0.761
	Slope	−1.764	0.082
bvFTD versus nfvPPA	Intercept	−0.220	0.827
	Slope	−1.787	0.078
svPPA versus nfvPPA	Intercept	−0.413	0.680
	Slope	0.188	0.852
Latent variable II			
bvFTD versus svPPA	Intercept	−1.412	0.162
	Slope	−0.426	0.672
bvFTD versus nfvPPA	Intercept	−2.575	**0**.012*
	Slope	−2.192	**0**.032*
svPPA versus nfvPPA	Intercept	−0.654	0.516
	Slope	−1.336	0.186

*P*-values are reported after correction for multiple comparisons using a false discovery rate controlling method with a significance threshold of 0.05. Significant intercept or slope differences are indicated by an asterisk.

bvFTD, behavioural variant FTD; nfvPPA, non-fluent variant PPA; svPPA, semantic variant PPA.

### Stability of brain atrophy and clinical patterns

To ensure that age, sex or education were not driving the observed relationship between MRI-based characteristics and cognitive function in the patients, we repeated the analyses after regressing out the effects of age, sex and education based on the healthy control group using *w*-scores. The results remained largely unchanged in terms of the number of LVs, their cognitive patterns and differences between FTD variants. This suggests that the findings in FTD patients reflect disease processes rather than changes in cognitive functioning in healthy aging, sex differences or effects of differences in education levels. DBM-based patterns were very similar after this step, as shown in Supplementary Fig. 4.

### Predictive capacity of the model

We examined the capacity of the combination of neurodegeneration and cognitive patterns in the LVs identified in the PLS analysis as features to predict clinical outcomes in patients. We applied a multi-class machine learning classifier with a 10-fold cross-validation loop on 100 randomized train and test splits. The predicted FTD subtype classification based on brain and cognition scores (including 16 cognitive scores and age, education and sex) was then compared with clinician variant diagnoses; the resulting three-class mean prediction accuracy over all repetitions was 89.12%. The mean sensitivity ranged from 84.19% for nfvPPA to 93.56% for bvFTD, with mean specificities of 91.46% for bvFTD to 97.15% for svPPA (Table 3). When we exclusively included brain scores in our model, the accuracy was reduced to 69.76%, whereas predictions based on the full battery of clinical scores were 86.05% accurate.

**Table 3:** Results of the classification analysis

	Classification accuracy	Sensitivity	Specificity	Balanced accuracy
Maximal model				
Brain + cognition (including 16 cognitive scores, age/sex/education and brain patterns)	89.12% (SD = 1.21%)	bvFTD: 88.88% (SD = 2.10%)	bvFTD: 91.46% (SD = 1.75%)	bvFTD: 90.17%
		svPPA: 93.56% (SD = 2.04%)	svPPA: 97.15% (SD = 1.06%)	svPPA: 95.36%
		nfvPPA: 84.19% (SD = 3.02%)	nfvPPA: 94.65% (SD = 0.80%)	nfvPPA: 89.42%
Cognition (including 16 cognitive scores, age/sex/education)	86.05% (SD = 1.51%)	bvFTD: 87.34% (SD = 2.49%)	bvFTD: 91.65% (SD = 1.63%)	bvFTD: 89.50%
		svPPA: 84.92% (SD = 2.12%)	svPPA: 94.53% (SD = 0.57%)	svPPA: 89.73%
		nfvPPA: 85.43% (SD = 2.61%)	nfvPPA: 92.74% (SD = 1.54%)	nfvPPA: 89.08%
Brain (including brain patterns and age/sex/education)	69.76% (SD = 2.73%)	bvFTD: 63.50% (SD = 4.28%)	bvFTD: 76.28% (SD = 3.15%)	bvFTD: 69.89%
		svPPA: 93.76% (SD = 2.00%)	svPPA: 94.92% (SD = 1.65%)	svPPA: 94.34%
		nfvPPA: 50.71% (SD = 6.51%)	nfvPPA: 82.47% (SD = 2.26%)	nfvPPA: 66.59%
Brain + cognition projection to longitudinal data	88.09% (SD = 1.45%)	bvFTD: 85.71% (SD = 3.43%)	bvFTD: 95.65% (SD = 0.86%)	bvFTD: 90.68%
		svPPA: 92.31% (SD = 1.56%)	svPPA: 95.83% (SD = 0.48%)	svPPA: 94.07%
		nfvPPA: 90.00% (SD = 0%)	nfvPPA: 92.59% (SD = 1.82%)	nfvPPA: 91.30%
Minimal model				
Brain + cognition (including CDR, BNT, age/sex/education and brain patterns)	83.62% (SD = 1.19%)	bvFTD: 84.35% (SD = 1.61%)	bvFTD: 86.38% (SD = 1.76%)	bvFTD: 85.37%
		svPPA: 87.50% (SD = 0.00%)	svPPA: 93.34% (SD = 0.79%)	svPPA: 90.42%
		nfvPPA: 76.17% (SD = 4.21%)	nfvPPA: 93.51% (SD = 0.89%)	nfvPPA: 84.84%
Brain + cognition sample included in maximal model	87.18% (SD = 1.11%)	bvFTD: 90.66% (SD = 0.31%)	bvFTD: 86.93% (SD = 1.89%)	bvFTD: 88.80%
		svPPA: 92.00% (SD = 0%)	svPPA: 94.34% (SD = 0%)	svPPA: 93.17%
		nfvPPA: 76.14% (SD = 4.14%)	nfvPPA: 98.26% (SD = 0.18%)	nfvPPA: 87.20%
Cognition (including CDR, BNT, age/sex/education)	76.38% (SD = 1.20%)	bvFTD: 84.82% (SD = 1.80%)	bvFTD: 80.91% (SD = 1.74%)	bvFTD: 82.87%
		svPPA: 70.19% (SD = 1.74%)	svPPA: 89.07% (SD = 0.82%)	svPPA: 79.63%
		nfvPPA: 61.13% (SD = 3.08%)	nfvPPA: 91.52% (SD = 1.23%)	nfvPPA: 76.33%
Cognition sample included in maximal model	76.54% (SD = 1.14%)	bvFTD: 88.69% (SD = 1.65%)	bvFTD: 81.22% (SD = 1.56%)	bvFTD: 84.95%
		svPPA: 69.84% (SD = 2.16%)	svPPA: 89.43% (SD = 1.04%)	svPPA: 79.64%
		nfvPPA: 65.90% (SD = 2.51%)	nfvPPA: 92.84% (SD = 0.92%)	nfvPPA: 79.37%
Brain + cognition projection to longitudinal data	85.77% (SD = 2.04%)	bvFTD: 81.77% (SD = 2.64%)	bvFTD: 94.43% (SD = 2.45%)	bvFTD: 88.10%
		svPPA: 92.85% (SD = 2.59%)	svPPA: 90.27% (SD = 1.33%)	svPPA: 91.56%
		nfvPPA: 87.00% (SD = 5.71%)	nfvPPA: 94.38% (SD = 1.72%)	nfvPPA: 90.69%

BNT, Boston Naming Test; bvFTD, behavioural variant FTD; CDR, Clinical Dementia Rating; nfvPPA, non-fluent variant PPA; svPPA, semantic variant PPA.

####  

##### Classification analysis with minimal input variables

To assess whether our model was adaptable for a clinical setting with limited time and resources, we repeated our analyses while only including the CDR scales and the BNT into the PLS and our classification model (Supplementary Fig. 5). Using these minimal variables, our model still achieved an accurate FTD subtype classification of 83.62%. Here, the combination of neurodegenerative and clinical measures was crucial as predictions only using the cognitive scores were only accurate 76.38% of the time. Notably, adding MRI measures to the clinical model increased the sensitivity for both PPAs by over 15% (70.19–87.50% for svPPA and 61.13–76.17% for nfvPPA).

When limiting the classification analysis to participants at the UCSF site to avoid confounding effects of site variability, the accuracy of the minimal model increased to 87.15% (brain and cognition) and 79.36% (cognition only). bvFTD patients at the UCSF site had significantly higher scores in all CDR subscales (see Supplementary Table 3), showing increased disease severity compared with bvFTD patients at the other two sites, suggesting that differences in the protocols or participant characteristics at the sites influenced model performance. Within the other FTD groups, only two svPPA patients were recruited at sites other than UCSF.

##### Validation of classification model

To ensure our models did not solely rely on disease severity as a predictor, we repeated all the analyses in severity-matched subsamples (CDR < 1.5). The models achieved similar accuracies within these severity-matched samples (88.88% for maximal model and 81.54% for minimal model; see Supplementary Table 9), suggesting that the models do not use severity in symptoms to differentiate the patients across variants. Similarly, the accuracy of our classification analysis in the age-matched sample was 88.74% for the maximal model and 83.78% for the minimal model, again suggesting that results are not merely driven by age differences in the groups.

Projecting the results of the PLS analysis onto the longitudinal data of the same cohort as an in-sample validation (with subject-level cross-validation), our maximal classification model achieved an accuracy of 88.09% and our minimal model was 85.77% accurate, suggesting reliability of both classifiers.

### Longitudinal progression of brain atrophy and clinical patterns

**Figure 5:**
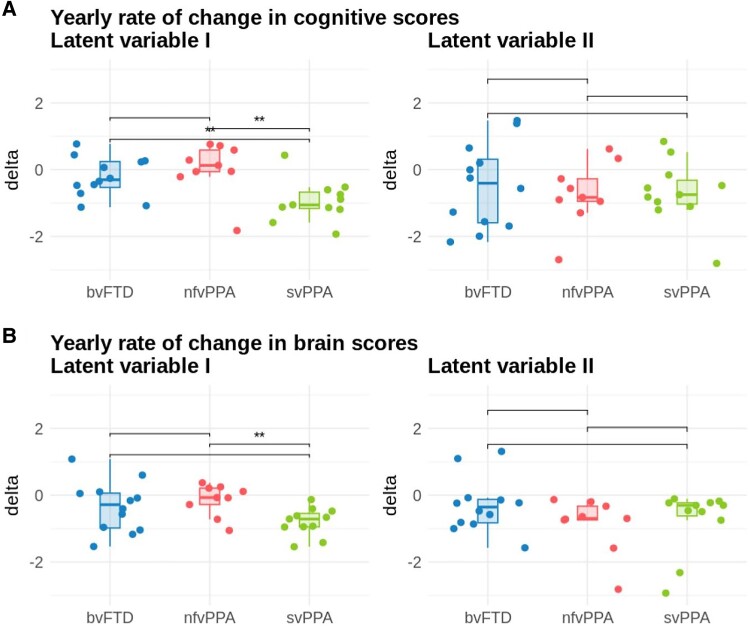
**Boxplots showing the longitudinal progression of brain and cognition scores for LVs I and II in three FTD subtypes.** Data points show the yearly rate of change in scores between the baseline and 1-year follow-up visit in FTD patients with longitudinal data, separated by FTD variant. Each data point represents one patient (*N* = 32). Asterisks indicate significant group differences based on unpaired *t*-tests with Tukey's test correction for multiple comparisons comparing the FTD groups. (**A**) Yearly rate of change in cognition scores in the three FTD variants (LV-I: svPPA versus bvFTD *tStat* = −2.85, *P* < 0.01, svPPA versus nfvPPA *tStat* = −3.13, *P* < 0.006). (**B**) Yearly rate of change in brain scores in the three FTD variants (LV-I: svPPA versus nfvPPA *tStat* = −3.30, *P* = 0.004). bvFTD, behavioural variant FTD; nfvPPA, non-fluent variant PPA; svPPA, semantic variant PPA.

In terms of the longitudinal changes of brain and cognition scores in the entire patient cohort, we found that the brain patterns in all four LVs (I: *P* = 0.007, II: *P* < 0.001, III: *P* < 0.001 and IV: *P* < 0.003) as well as cognition patterns in LVs I and II (I: *P* = 0.006 and II: *P* < 0.002) significantly worsened over time (Supplementary Fig. 6). Scores in the cognitive pattern of LV-IV slightly increased over time (*P* = 0.007). As expected, since baseline and follow-up visits were only ∼1 year apart, the changes are relatively small. With regard to the yearly rate of change in the three diagnostic groups, we only found differences between variants in LV-I (Fig. 5). Here, we found significant differences in the delta values for cognitive scores, with increased changes in svPPA individuals compared with both bvFTD (*tStat* = −2.85, *P* < 0.01) and nfvPPA (*tStat* = −3.13, *P* < 0.006). Similarly, the svPPA variant changed more in terms of their brain scores than the nfvPPA subtype (*tStat* = −3.30, *P* = 0.004).

## Discussion

The present study links clinical features of the three core FTD subtypes, bvFTD, svPPA and nfvPPA, to the underlying brain atrophy pattern using a single integrated analysis. In these three cohorts, in addition to higher age, a range of cognitive characteristics, including global cognitive function, language and executive function, were linked to brain atrophy. While atrophy patterns were widely distributed across both cortical and subcortical structures, the covariance between atrophy and cognitive measures was largely explained by the involvement of frontal and temporal lobes and subcortical structures. We also found differences in the relationship between atrophy and cognition between bvFTD, svPPA and nfvPPA. This allowed us to use the observed atrophy and cognition patterns in a prediction model, yielding high prediction accuracy (89.12%), sensitivity and specificity in a three-way classification of FTD patients into phenotypes as compared with clinician subtype diagnosis as a gold standard. Even when only including minimal features (DBM values, CDR subscales and BNT scores) as predictors, our model outperformed previous attempts at automated diagnosis of FTD, thereby demonstrating clinical utility. In this scenario, the MRI-derived features crucially contributed to the diagnostic accuracy (76% versus 83% without and with MRI features, respectively). This provides promising evidence that the combination of DBM and multivariate statistical methods could potentially aid the automated diagnosis and classification of FTD patients. In the clinic, our tool has potential utility in non-specialized clinics or in the case of restricted access to specialized clinicians or memory clinics. In these contexts, it could aid in determining the probable FTD subtype of a patient and ensure they receive appropriate initial treatment, particularly support through speech and language or psychotherapy. As our minimal model solely requires MRI scans and two comparatively short tests, this is an accessible approach.

Our data-driven PLS results align with previous findings on neuropathological changes in FTD. PLS is a multivariate statistical method that examines the relationships between two sets of variables (i.e. regional brain atrophy and cognitive performance measures) by identifying underlying components that capture the maximum covariance between them. This is achieved by identifying a linear combination of variables for each set of variables for each LV that ensures the maximum covariation. In other words, identifying a linear combination of regional brain atrophy patterns can explain a linear combination of cognitive performances. This is akin to a data-driven approach to identify a ‘brain network’ that is related to a cognitive ‘domain’ as measured by our clinical tests. In our case, PLS resulted in four sets of brain atrophy patterns and associated patterns of cognitive performance. We found that the covariation of FTD groups explained in LV-I was partially explained by atrophy in the temporal lobe. While temporal lobe atrophy has been mostly associated with svPPA,^13^ some studies indicate that it can also contribute to symptoms in bvFTD, especially in combination with hippocampal abnormalities,^58^ which we also saw in LV-II. This has been interpreted as the involvement of dysfunction of the default mode, limbic and salience networks in the pathogenesis of bvFTD,^59^ leading to the observed changes in emotional and social processing. Particularly in LV-II, our results show that atrophy in the frontal lobe plays a crucial role in behavioural and cognitive symptoms in FTD, including impairment of executive function, attention and language. Additionally, we found that structural changes in the insula contributed to cognitive decline and specifically seemed to distinguish nfvPPA from svPPA in LV-I. Due to its extensive anatomical and functional connections to linguistic, motor and sensory areas in the frontal cortex,^60,61^ the insula has been associated with semantic, syntactic and phonological processing^62^ and seems to be involved in the development of speech apraxia in nfvPPA.^24,63^ The atrophy pattern in LV-III was largely driven by cerebellar structural changes. Behavioural impairment in genetic FTD has been associated with cerebellar atrophy^58,64^ and seems to show distinct patterns between FTD subtypes, specifically involvement of lobule VII in bvFTD and lobule VI in nfvPPA,^65^ which we found in LV-I, although we did not find focal atrophy in lobule V as previously described.^65^ While the contribution of frontotemporal lobar degeneration to cognitive symptoms in FTD has been widely established,^5^ subcortical atrophy is only recently receiving more attention. In LV-II, we found pronounced atrophy around the ventricles as distinctive features between FTD subgroups, confirming findings that ventricular expansion is a common feature of bvFTD and constitutes a sensitive and reliable marker of disease progression.^34^ Involvement of the thalamus and amygdala, which we found in several LVs, has been reported for both sporadic and genetic FTD and seems to precede cortical atrophy.^58,59,66^ Previous studies have shown distinctive atrophy patterns in the thalamus between the core FTD subtypes.^67,68^ Considering that the amygdala is part of the limbic system and is implicated in emotional processing and reward learning,^69^ it is not surprising that our analyses found involvement of this structure. Overall, this suggests that investigating subcortical atrophy in FTD is a promising avenue for both diagnostic purposes and improving our understanding of neural mechanisms underlying clinical presentation. It also highlights the utility of DBM in assessing cortical and subcortical atrophy given its improved accuracy in detecting structural changes in deeper brain layers.

To test whether the brain and cognition patterns derived from the PLS analysis were useful as informative features that could separate individual patients with different FTD syndromes, we assessed prediction of FTD subtype in an automated machine learning procedure. Classification accuracy was 89.12%, with sensitivities for different variants ranging from 84.19% to 93.56% and specificities of 91.46–97.15%. We validated the robustness of our model by testing our model in a disease severity-matched and an age-matched sample of this cohort and obtained similar accuracies. Additionally, we used the longitudinal data of our cohort as an in-sample validation test set in the cross-validated classification analysis to determine whether our classifier would also predict FTD subtypes in later disease stages. Importantly, we also tested whether the combination of atrophy scores and only two neuropsychological tests (CDR and BNT) would be sufficient to distinguish FTD syndromes. These measures were chosen because they are clinically useful and because they require little time compared with the full test battery used in our maximal model. This is relevant as patients’ attention and capability to endure lengthy examinations are reduced and clinicians have limited time for extensive testing. Even with these minimal input variables (combined with the MRI information), our model achieved an accuracy of 83.62%. These accuracies are comparable with or superior to those of similar studies. While studies usually achieve high accuracy when differentiating FTD patients from healthy controls,^11^ few groups attempted to identify the FTD subtype.^23,42^ The only study that used a three-way comparison rather than comparing two groups against each other at a time resulted in high specificity (94.2–97.5%) but low sensitivity (0–50%) in identifying one FTD subtype from all others.^43^ Given that predictions were less accurate in our study when we used cognitive variables only, the strength of our approach seems to lie in the combination of neurodegenerative and clinical characteristics in the patients, particularly the use of DBM. While a previous study by Manera *et al*.^70^ achieved similar accuracy in differentiating bvFTD from other FTD variants based on ventricular features estimated via DBM, our model outperforms this approach due to our three-way classifier (with balanced accuracies of 66% for bvFTD, 69% for svPPA and 94% for nfvPPA when only using DBM patterns as input to the prediction model) as opposed to this study’s two-way classifications (with 66% for bvFTD versus svPPA and 71% for bvFTD versus nfvPPA). Moreover, the minimal model's performance improved significantly when the analysis was restricted to participants from the primary FTLDNI research site (UCSF), achieving an accuracy comparable with that of the maximal model (87.15%). This improvement might partially be attributable to the elimination of inter-site variability in MRI scanners and protocols. However, since the cognition-only model also showed similar levels of increase in performance with UCSF participants (reaching 79.36% accuracy overall and up to a 5% improvement in balanced accuracies), this difference is most likely due to differences in participant characteristics, and not MRI features. Given that bvFTD patients at UCSF exhibit distinct clinical characteristics and greater disease severity relative to other sites, it is likely that these participant characteristics contributed to the observed differences in model performance. This further suggests that the increased accuracy of the maximal model was influenced not solely by the addition of cognitive scores but also by these site-specific factors. Consequently, our secondary minimal model that was evaluated using the same data set achieves performance comparable with the maximal model, further supporting the combined use of DBM measures and minimal cognitive assessments for effective subtyping of FTD. Another advantage of our study is the application of a multivariate statistics method that allows us to capture the maximal covariance across features that leads to a data-driven separation between groups, indicated by the clear separation between variants in terms of brain scores in LVs I and IV particularly. These results suggest that automated methods incorporating DBM-derived atrophy and clinical performance could assist in the diagnosis of FTD subtypes.

One of the strengths of this study is that our image processing pipelines have been developed and extensively validated for use in multi-centre and multi-scanner data sets of aging and neurodegenerative disease populations and provide robust and sensitive DBM measures.^32,34,54^ We further quality controlled all the steps of the pipeline to ensure the accuracy of the results. Similarly, we have demonstrated the reliability of our analysis results repeating all steps after regressing out the effects of healthy aging. An intriguing result of this study is the high prediction accuracy (over 89%) our approach achieved for classifying FTD patients according to FTD variant, with similar results in a classification model that solely included two short clinical tests and brain atrophy measures. This is particularly relevant given that automated subtype diagnosis of FTD still poses a challenge, despite advances in machine learning and neuroimaging.^11^ This highlights the increased sensitivity of DBM as well as the potential utility of DBM paired with multivariate statistics in the diagnosis of FTD. This is an important step for improving patient care and diagnostic prognosis.

The present study has some limitations that need to be taken into account. Although PLS provides a comprehensive approach to investigating brain–cognition relations, it cannot provide insight into how each particular clinical manifestation potentially relates to a specific brain region, rather than the atrophy pattern as a whole. Such individual relationships need to be addressed in future studies. Another methodological consideration is that these results are valid only for the sample of FTD patients included here. Further validation is needed in larger, more diverse samples before we can be confident that the observed results will generalize to the rest of the population. These samples should include logopenic and semantic behavioural as well as genetic variants of FTD to cover the entire disease spectrum as well as participants with a higher range of educational and ethnic backgrounds. Furthermore, sample sizes in the FTLDNI cohort are small, particularly for the two PPA groups, and some relevant information on the participants is missing, such as disease duration, time from diagnosis and comorbidities, potentially obscuring confounding factors in our analyses. It should also be noted that the bvFTD patients displayed higher disease severity than other groups, which might impact our analyses. Likewise, our analyses could not cover all cognitive domains due to high numbers of missing values in some tests included in the data set (e.g. only 44% of participants underwent the Modified Benson Figure Test for visuospatial ability). In addition, the FTLDNI did not collect information on cognitive areas like complex attention and specific skills like language comprehension and writing/reading skills, for instance. To investigate the diagnostic utility of our approach that uses machine learning and multivariate statistics, future studies could directly compare traditional approaches, including cortical thickness and voxel-based morphometry, to our results and include genetic variants of FTD in their sample to ensure generalizability over the entire spectrum of FTD. Similarly, it would be valuable to test our model in a sample of FTD patients with post-mortem histology information to assess whether it could predict underlying pathology as a non-invasive, lower-cost tool *in vivo*.

Altogether, findings in this study demonstrate a robust mapping between neurodegeneration as estimated by DBM values and the cognitive manifestations of the core FTD subtypes. The combination of DBM and multivariate statistical methods could potentially serve as an imaging biomarker for diagnosis and phenotyping in FTD and thereby improve early disease management and automated diagnosis.

## Supplementary Material

fcaf065_Supplementary_Data

## Data Availability

FTLDNI MRI and clinical measures are available through https://ida.loni.usc.edu/login.jsp. Derived data supporting the findings of this study, including individual PLS scores, are available from the corresponding author on request. Codes are available on GitHub (https://github.com/ameliemetz/FTD_subtype_prediction).
