# Supplementary material for: Frontotemporal dementia subtyping using machine learning, multivariate statistics and neuroimaging
Source: Brain Commun. 2025 Feb 11;7(1):fcaf065. doi: 10.1093/braincomms/fcaf065 (PMC11844796; doi:10.1093/braincomms/fcaf065)
Supplement: fcaf065_Supplementary_Data [file fcaf065_supplementary_data.docx]

**Supplementary Materials**

“Frontotemporal Dementia Subtyping using Machine Learning, Multivariate Statistics, and Neuroimaging”

Amelie Metz, Yashar Zeighami, Simon Ducharme, Sylvia Villeneuve, and Mahsa Dadar

**Supplementary Table 1. Number of participants recruited, scanned, and tested at each of the FTLDNI research sites.**

|  | **controls** | **bvFTD** | **svPPA** | **nfvPPA** |
| --- | --- | --- | --- | --- |
| *Mayo* | 9 | 15 | 1 | 0 |
| *MGH* | 0 | 4 | 2 | 1 |
| *UCSF* | 124 | 51 | 33 | 29 |

3.0 T MRIs were acquired at three sites [T1-weighted magnetization-prepared rapid gradient-echo imaging (MPRAGE)] using Siemens Trio Total imaging matrix (Tim) scanners, with the following parameters:

- UCSF: repetition time (TR) = 2.3 ms, echo time = 2.98 ms, inversion time = 900 ms, flip angle 9°, matrix 256 × 240, slice thickness 1 mm, voxel size 1 mm3
- Mayo: repetition time (TR) = 2.3 ms, echo time = 3.04 ms, inversion time = 900 ms, flip angle 8°, matrix 256 × 240, slice thickness 1.2 mm, voxel size 1 mm3
- MGH: repetition time (TR) = 2.3 ms, echo time = 2.96 ms, inversion time = 900 ms, flip angle 9°, matrix 256 × 240, slice thickness 1 mm, voxel size 1 mm3

The FTLDNI uses the infrastructure established by the Alzheimer's Disease Neuroimaging Initiative (ADNI). All participating imaging centers share a common platform.

**Supplementary Table 2. Baseline demographic and cognitive characteristics in healthy control group,** used for regressing out the effects of age, sex, and education (w-scores). Values expressed as mean (standard deviation).

|  | **Healthy controls** |
| --- | --- |
| **Demographics** | |
| *Number of participants* | 133 |
| *Age (years)* | 63.58 ± 7.54 [64.00] |
| *Sex (M:F, %M)* | 56:77 (42,11%) |
| *Education (years)* | 17.51 ± 1.76 [18.00] |
| **Cognition** | |
| *Clinical Dementia Rating sum of boxes* | 0.04 ± 0.16 [0.00] |
| *Clinical Dementia Rating language subscale* | 0.01 ± 0.06 [0.00] |
| *Clinical Dementia Rating behavior subscale* | 0.01 ± 0.08 [0.00] |
| *Mini-Mental Status Examination* | 29.40 ± 0.80 [30.00] |
| *California Verbal Learning Test memory* | 29.63 ± 3.89 [30.00] |
| *California Verbal Learning Test recall after 30second delay* | 8.01 ± 1.21 [9.00] |
| *California Verbal Learning Test recall after 10minute delay* | 7.79 ± 1.27 [8.00] |
| *California Verbal Learning Test recognition* | 8.27 ± 1.92 [9.00] |
| *Digit span forward* | 7.26 ± 1.20 [7.00] |
| *Digit span backward* | 5.47 ± 1.47 [5.00] |
| *Modified Trail Making Test correct lines* | 13.73 ± 1.77 [14.00] |
| *Modified Trail Making Test time* | 27.78 ± 14.64 [25.00] |
| *Verbal fluency phonological* | 16.29 ± 4.58 [16.00] |
| *Verbal fluency semantic* | 23.92 ± 5.30 [24.00] |
| *Boston Naming Test* | 14.59 ± 0.73 [15.00] |
| *Peabody Picture Vocabulary Test* | 15.79 ± 0.45 [16.00] |

**Supplementary Table 3. Baseline demographic and cognitive characteristics in FTD subtypes at different research sites**. This table exclusively includes participants included in the minimal model. Values are expressed as mean (standard deviation). Asterisks indicate significant group differences based on one-way ANOVA or chi-square analysis comparing the groups. bvFTD = behavioral variant FTD; svPPA = semantic variant primary progressive aphasia; nfvPPA = nonfluent variant primary progressive aphasia.

|  | **bvFTD** | | | **nfvPPA** | **svPPA** | | | **p-value** |
| --- | --- | --- | --- | --- | --- | --- | --- | --- |
|  | **Mayo** | **MGH** | **UCSF** | **UCSF** | **Mayo** | **MGH** | **USCF** |  |
| **Demographics** | | | | | | | |  |
| *Number of participants (total n=136)* | 15 | 4 | 46 | 23 | 1 | 1 | 30 |  |
| *Age (years)* | 62.8 (4.0) | 64.2 (2.2) | 60.6 (7.2) | 68.3 (8.5) | 69.0 (NA) | 64.0 (NA) | 62.9 (6.6) | 0.005 |
| *Sex (M:F, %M)* | 11:4 (73.3) | 4:0 (100.0) | 28:18 (60.9) | 11:12 (47.8) | 1:0 (100.0) | 0:1 (0.0) | 16:14 (53.3) | 0.249 |
| *Education (years)* | 15.8 (0.0) | 15.8 (0.1) | 15.9 (3.3) | 17.1 (3.5) | 17.4 (NA) | 17.4 (NA) | 16.9 (2.6) | 0.568 |
| **Cognition** | | | | | | | |  |
| *Clinical Dementia Rating sum of boxes* | 4.4 (3.1) | 4.2 (4.3) | 6.8 (2.9) | 2.2 (2.2) | 2.5 (NA) | 5.5 (NA) | 3.9 (2.2) | <0.001 |
| *Clinical Dementia Rating language subscale* | 0.3 (0.2) | 0.8 (0.8) | 0.8 (0.5) | 1.1 (0.7) | 0 (NA) | 2 (NA) | 1.0 (0.5) | <0.001 |
| *Clinical Dementia Rating behavior subscale* | 0.4 (0.3) | 0.8 (0.5) | 1.7 (0.7) | 0.5 (0.4) | 0.5 (NA) | 1.0 (NA) | 1.1 (0.7) | <0.001 |
| *Mini-Mental Status Examination* | 24.4 (6.2) | 23.0 (6.8) | 23.4 (4.5) | 25.3 (5.2) | 26.0 (NA) | 19.0 (NA) | 24.5 (5.2) | 0.743 |
| *California Verbal Learning Test memory* | 18.5 (7.6) | 18.0 (13.3) | 19.9 (6.7) | 22.2 (7.7) | 12.0 (NA) | 7.0 (NA) | 17.5 (6.4) | 0.134 |
| *California Verbal Learning Test recall after 30second delay* | 3.2 (3.0) | 4.5 (3.1) | 4.6 (2.4) | 5.6 (2.9) | 0.0 (NA) | 1.0 (NA) | 3.1 (2.5) | 0.006 |
| *California Verbal Learning Test recall after 10minute delay* | 1.5 (2.4) | 2.7 (2.5) | 3.7 (2.8) | 5.3 (2.9) | 0.0 (NA) | 0.0 (NA) | 2.0 (2.3) | <0.001 |
| *California Verbal Learning Test recognition* | 6.7 (2.4) | 7.5 (0.6) | 7.2 (1.7) | 8.2 (0.9) | 0.0 (NA) | 5.0 (NA) | 6.7 (2.1) | <0.001 |
| *Digit span forward* | 5.5 (1.3) | 5.8 (1.7) | 5.5 (1.2) | 5.0 (1.3) | 8.0 (NA) | 5.0 (NA) | 6.8 (1.5) | <0.001 |
| *Digit span backward* | 3.7 (1.1) | 4.5 (2.5) | 3.3 (1.3) | 3.5 (1.4) | 5.0 (NA) | 4.0 (NA) | 5.0 (1.3) | <0.001 |
| *Modified Trail Making Test correct lines* | 8.9 (5.8) | 11.5 (5.0) | 10.2 (4.8) | 12.0 (4.0) | 14.0 (NA) | 14.0 (NA) | 13.3 (2.6) | 0.034 |
| *Modified Trail Making Test time* | 3.6 (4.2) | 1.0 (0.8) | 1.9 (3.0) | 1.1 (1.7) | 0.0 (NA) | 0.0 (NA) | 0.5 (1.3) | 0.015 |
| *Verbal fluency phonological* | 6.2 (3.7) | 8.0 (5.4) | 6.6 (4.8) | 7.1 (5.0) | 7.0 (NA) | 0.0 (NA) | 9.0 (4.4) | 0.227 |
| *Verbal fluency semantic* | 8.6 (6.1) | 10.0 (6.4) | 9.6 (6.5) | 11.9 (8.6) | 3.0 (NA) | 3.0 (NA) | 8.5 (4.0) | 0.415 |
| *Boston Naming Test* | 12.1 (3.7) | 11.2 (1.0) | 12.3 (3.0) | 12.2 (2.9) | 2.0 (NA) | 1.0 (NA) | 5.3 (3.6) | <0.001 |
| *Peabody Picture Vocabulary Test* | NA | NA | 13.3 (3.3) | 14.5 (2.0) | NA | NA | 8.6 (4.2) | <0.001 |

**Supplementary Table 4. Baseline demographic and cognitive characteristics in FTD subtypes**. Significance levels were determined through independent t-tests with Tukey HSD correction for multiple comparisons. Asterisks indicate significant group differences (p<0.05). Asterisks indicate significant group differences based on independent t-tests with Tukey HSD correction for multiple comparisons. bvFTD = behavioral variant FTD; svPPA = semantic variant primary progressive aphasia; nfvPPA = nonfluent variant primary progressive aphasia.

| **Variable** | **Comparison** | **p-value** |
| --- | --- | --- |
| *Age* | nfvPPA vs. bvFTD | **<0.001*** |
|  | svPPA vs. bvFTD | 0.511 |
|  | svPPA vs. nfvPPA | **0.007*** |
| *Education* | nfvPPA vs. bvFTD | 0.118 |
|  | svPPA vs. bvFTD | 0.056 |
|  | svPPA vs. nfvPPA | 0.984 |
| *CDR box score* | nfvPPA vs. bvFTD | **<0.001*** |
|  | svPPA vs. bvFTD | **<0.001*** |
|  | svPPA vs. nfvPPA | 0.074 |
| *CDR language* | nfvPPA vs. bvFTD | **<0.001*** |
|  | svPPA vs. bvFTD | **0.025*** |
|  | svPPA vs. nfvPPA | 0.441 |
| *CDR behavior* | nfvPPA vs. bvFTD | **<0.001*** |
|  | svPPA vs. bvFTD | 0.229 |
|  | svPPA vs. nfvPPA | **0.004*** |
| *CVLT recall after 30second delay* | nfvPPA vs. bvFTD | 0.132 |
|  | svPPA vs. bvFTD | **0.049*** |
|  | svPPA vs. nfvPPA | **0.001*** |
| *CVLT recall after 10minute delay* | nfvPPA vs. bvFTD | **0.006*** |
|  | svPPA vs. bvFTD | 0.056 |
|  | svPPA vs. nfvPPA | **<0.001*** |
| *CVLT recognition* | nfvPPA vs. bvFTD | **0.040*** |
|  | svPPA vs. bvFTD | 0.181 |
|  | svPPA vs. nfvPPA | **0.001*** |
| *Digit span forward* | nfvPPA vs. bvFTD | 0.191 |
|  | svPPA vs. bvFTD | **<0.001*** |
|  | svPPA vs. nfvPPA | **<0.001*** |
| *Digit span backward* | nfvPPA vs. bvFTD | 0.999 |
|  | svPPA vs. bvFTD | **<0.001*** |
|  | svPPA vs. nfvPPA | **<0.001*** |
| *MTMT correct lines* | nfvPPA vs. bvFTD | 0.081 |
|  | svPPA vs. bvFTD | **0.002*** |
|  | svPPA vs. nfvPPA | 0.577 |
| *MTMT time* | nfvPPA vs. bvFTD | 0.449 |
|  | svPPA vs. bvFTD | **0.006*** |
|  | svPPA vs. nfvPPA | 0.275 |
| *Verbal fluency phonological* | nfvPPA vs. bvFTD | 0.947 |
|  | svPPA vs. bvFTD | 0.114 |
|  | svPPA vs. nfvPPA | 0.376 |
| *PPVT* | nfvPPA vs. bvFTD | 0.277 |
|  | svPPA vs. bvFTD | **<0.001*** |
|  | svPPA vs. nfvPPA | **<0.001*** |

**Supplementary Table 5. Missing values in all variables at baseline and 1-year follow-up visits.**

| **Variable** | **Number and percentage of missing values per group and visit** | | | | | | |
| --- | --- | --- | --- | --- | --- | --- | --- |
|  | controls | bvFTD | | svPPA | | nfvPPA | |
|  | baseline | baseline | 1-year follow-up | baseline | 1-year follow-up | baseline | 1-year follow-up |
| *DBM baseline* | 16 excluded due to low image quality/failed visual QC | | | | | | |
| Total number of participants | 133 | 70 | 48 | 36 | 30 | 30 | 21 |
| Education in years | 16 (12.03%) | 18 (25.71%) | 13 (27.08%) | 3 (8.33%) | 3 (10%) | 2 (6.67%) | 1 (4.76%) |
| *Clinical Dementia Rating sum of boxes* | 73 (54.89%) | 2 (2.86%) | 1 (2.08%) | 2 (5.56%) | 2 (6.67%) | 3 (10%) | 0 (0%) |
| *Clinical Dementia Rating language subscale* | 73 (54.89%) | 4 (5.71%) | 1 (2.08%) | 2 (5.56%) | 2 (6.67%) | 3 (10%) | 0 (0%) |
| *Clinical Dementia Rating behavior subscale* | 68 (51.13%) | 2 (2.86%) | 0 (0%) | 2 (5.56%) | 2 (6.67%) | 3 (10%) | 0 (0%) |
| *Mini-Mental Status Examination* | 15 (11.28%) | 7 (10%) | 4 (8.33%) | 3 (8.33%) | 4 (13.33%) | 6 (20%) | 3 (14.29%) |
| *California Verbal Learning Test memory* | 66 (49.62%) | 7 (10%) | 4 (8.33%) | 7 (19.44%) | 8 (26.67%) | 8 (26.67%) | 5 (23.81%) |
| *California Verbal Learning Test recall after 30second delay* | 66 (49.62%) | 9 (12.86%) | 4 (8.33%) | 7 (19.44%) | 8 (26.67%) | 8 (26.67%) | 5 (23.81%) |
| *California Verbal Learning Test recall after 10minute delay* | 66 (49.62%) | 11 (15.71%) | 4 (8.33%) | 8 (22.22%) | 9 (30%) | 8 (26.67%) | 5 (23.81%) |
| *California Verbal Learning Test recognition* | 66 (49.62%) | 13 (18.57%) | 4 (8.33%) | 8 (22.22%) | 9 (30%) | 8 (26.67%) | 5 (23.81%) |
| *Modified Trail Making Test correct lines* | 15 (11.28%) | 23 (32.86%) | 13 (27.08%) | 10 (27.78%) | 8 (26.67%) | 8 (26.67%) | 7 (33.33%) |
| *Modified Trail Making Test time* | 15 (11.28%) | 21 (30%) | 12 (25%) | 9 (25%) | 7 (23.33%) | 8 (26.67%) | 7 (33.33%) |
| *Digit span forward* | 19 (14.29%) | 9 (12.86%) | 4 (8.33%) | 6 (16.67%) | 6 (20%) | 7 (23.33%) | 6 (28.57%) |
| *Digit span backward* | 16 (12.03%) | 9 (12.86%) | 5 (10.42%) | 6 (16.67%) | 5 (16.67%) | 9 (30%) | 6 (28.57%) |
| *Verbal fluency phonological* | 28 (21.05%) | 8 (11.43%) | 7 (14.58%) | 9 (25%) | 8 (26.67%) | 7 (23.33%) | 5 (23.81%) |
| *Verbal fluency semantic* | 16 (12.03%) | 8 (11.43%) | 5 (10.42%) | 8 (22.22%) | 7 (23.33%) | 7 (23.33%) | 6 (28.57%) |
| *Boston Naming Test* | 17 (12.78%) | 7 (10%) | 5 (10.42%) | 5 (13.89%) | 5 (16.67%) | 7 (23.33%) | 4 (19.05%) |
| *Peabody Picture Vocabulary Test* | 89 (66.92%) | 30 (42.86%) | 24 (50%) | 10 (27.78%) | 15 (50%) | 8 (26.67%) | 7 (33.33%) |

**
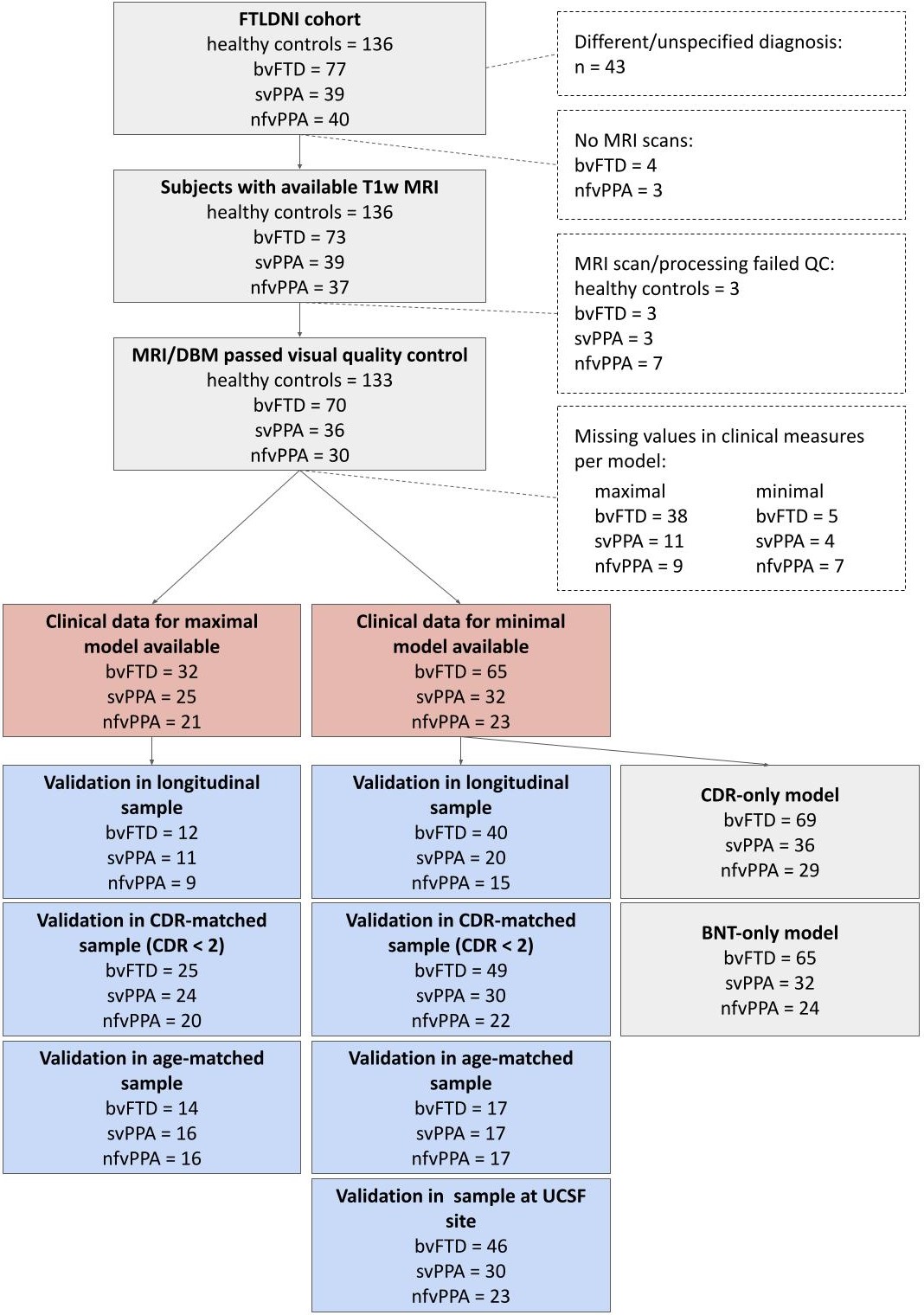
**

**Supplementary Figure 1. Flowchart of the sample composition at each step of the analyses.**

**Supplementary Table 6. CerebrA regions that contributed significantly to latent variables in the PLS analysis, as estimated by bootstrapping values (p < 0.05) and confidence intervals.**

| **Latent variable** | **CI lower** | **CI upper** | **Hemisphere** | **Region** | **CerebrA structure** |
| --- | --- | --- | --- | --- | --- |
| **LV-I** | -0.180 | -0.859 | Left | Subcortical | inferior_lateral_ventricle |
|  | -0.014 | -0.582 | Left | Cerebellum | cerebellum_white_matter |
|  | -0.038 | -0.517 | Left | Cerebellum | cerebellum_exterior |
|  | -0.034 | -0.506 | Right | Cerebellum | cerebellar_vermal_lobules_VI-VII |
|  | -0.001 | -0.528 | Left | Nerve | optic_chiasm |
|  | -0.019 | -0.507 | Right | Nerve | optic_chiasm |
|  | -0.001 | -0.517 | Right | Subcortical | inferior_lateral_ventricle |
|  | -0.024 | -0.491 | Right | Cerebellum | cerebellum_exterior |
|  | -0.001 | -0.443 | Left | Cerebellum | cerebellar_vermal_lobules_VI-VII |
|  | -0.015 | -0.408 | Right | Cerebellum | cerebellar_vermal_lobules_IV |
|  | 0.874 | 0.228 | Left | Temporal lobe | inferior_temporal |
|  | 0.876 | 0.205 | Left | Subcortical | amygdala |
|  | 0.817 | 0.199 | Left | Temporal lobe | fusiform |
|  | 0.780 | 0.234 | Left | Temporal lobe | middle_temporal |
|  | 0.763 | 0.140 | Left | Frontal lobe | medial_orbitofrontal |
|  | 0.716 | 0.163 | Left | Insula | insula |
|  | 0.688 | 0.158 | Left | Temporal lobe | superior_temporal |
|  | 0.657 | 0.142 | Left | Subcortical | accumbens_area |
|  | 0.627 | 0.125 | Right | Subcortical | amygdala |
|  | 0.619 | 0.111 | Left | Temporal lobe | entorhinal |
|  | 0.607 | 0.071 | Left | Subcortical | parahippocampal |
|  | 0.574 | 0.096 | Right | Temporal lobe | fusiform |
|  | 0.542 | 0.094 | Right | Temporal lobe | inferior_temporal |
|  | 0.540 | 0.090 | Left | Subcortical | basal_forebrain |
|  | 0.497 | 0.094 | Right | Temporal lobe | entorhinal |
|  | 0.529 | 0.058 | Right | Frontal lobe | pars_orbitalis |
|  | 0.551 | 0.027 | Left | Cingulate | caudal_anterior_cingulate |
|  | 0.499 | 0.055 | Right | Cingulate | rostral_anterior_cingulate |
|  | 0.505 | 0.023 | Left | Cingulate | isthmus_cingulate |
| **LV-II** | -0.176 | -0.739 | Left | Subcortical | lateral_ventricle |
|  | -0.085 | -0.758 | Right | Subcortical | 3rd_ventricle |
|  | -0.124 | -0.674 | Right | Subcortical | lateral_ventricle |
|  | -0.064 | -0.715 | Left | Subcortical | 3rd_ventricle |
|  | -0.101 | -0.609 | Left | Temporal lobe | entorhinal |
|  | -0.057 | -0.580 | Left | Subcortical | hippocampus |
|  | -0.030 | -0.531 | Right | Subcortical | amygdala |
|  | -0.003 | -0.453 | Left | Subcortical | basal_forebrain |
|  | 0.665 | 0.145 | Left | Frontal lobe | precentral |
|  | 0.666 | 0.107 | Left | Frontal lobe | caudal_middle_frontal |
|  | 0.629 | 0.109 | Left | Frontal lobe | rostral_middle_frontal |
|  | 0.640 | 0.097 | Left | Subcortical | pallidum |
|  | 0.615 | 0.115 | Left | Frontal lobe | precentral |
|  | 0.621 | 0.069 | Right | Frontal lobe | caudal_middle_frontal |
|  | 0.578 | 0.082 | Left | Frontal lobe | pars_opercularis |
|  | 0.624 | 0.036 | Left | Cingulate | posterior_cingulate |
|  | 0.581 | 0.069 | Right | Subcortical | pallidum |
|  | 0.504 | 0.093 | Left | Frontal lobe | superior_frontal |
|  | 0.533 | 0.040 | Left | Frontal lobe | pars_orbitalis |
|  | 0.496 | 0.074 | Left | Subcortical | thalamus |
|  | 0.532 | 0.013 | Right | Frontal lobe | rostral_middle_frontal |
|  | 0.494 | 0.023 | Right | Frontal lobe | lateral_orbitofrontal |
|  | 0.459 | 0.043 | Right | Subcortical | thalamus |
|  | 0.446 | 0.046 | Right | Frontal lobe | superior_frontal |
|  | 0.445 | 0.029 | Right | Temporal lobe | transverse_temporal |
|  | 0.454 | 0.006 | Left | Frontal lobe | precuneus |
|  | 0.390 | 0.026 | Left | Subcortical | ventral_DC |
|  | 0.368 | 0.007 | Right | Parietal lobe | superior_parietal |
| **LV-III** | -0.103 | -0.675 | Left | Frontal lobe | lateral_orbitofrontal |
|  | -0.111 | -0.631 | Left | Frontal lobe | rostral_middle_frontal |
|  | -0.071 | -0.470 | Right | Frontal lobe | superior_frontal |
|  | -0.052 | -0.395 | Right | Subcortical | parahippocampal |
|  | 0.542 | 0.063 | Right | Cerebellum | cerebellum_white_matter |
|  | 0.420 | 0.056 | Left | Subcortical | ventral_DC |
|  | 0.400 | 0.007 | Right | Cerebellum | cerebellar_vermal_lobules_IV |
| **LV-IV** | -0.175 | -0.621 | Left | Subcortical | caudate |
|  | -0.016 | -0.526 | Right | Subcortical | caudate |
|  | 0.540 | 0.188 | Right | Subcortical | basal_forebrain |
|  | 0.555 | 0.132 | Left | Cerebellum | cerebellar_vermal_lobules_IV |
|  | 0.517 | 0.050 | Right | Cerebellum | cerebellum_exterior |
|  | 0.520 | 0.041 | Left | Cerebellum | cerebellum_exterior |
|  | 0.503 | 0.053 | Left | Subcortical | basal_forebrain |
|  | 0.504 | 0.023 | Left | Cingulate | isthmus_cingulate |
|  | 0.463 | 0.048 | Right | Subcortical | thalamus |
|  | 0.431 | 0.027 | Right | Temporal lobe | entorhinal |
|  | 0.437 | 0.020 | Right | Subcortical | 4th_ventricle |
|  | 0.422 | 0.022 | Right | Frontal lobe | superior_frontal |
|  | 0.392 | 0.003 | Right | Subcortical | accumbens_area |

**Supplementary Table 7. Contribution of clinical scores to latent variables in the PLS analysis, as estimated by bootstrapping values (p < 0.05) and confidence intervals. Clinical test scores that contributed significantly based on Confidence Intervals are highlighted in bold.**

| **Latent variable** | **CI lower** | **CI upper** | **Test** |
| --- | --- | --- | --- |
| **LV-I** | 0.769 | 0.516 | **BNT** |
|  | 0.711 | 0.509 | **CVLT 10m delay** |
|  | 0.708 | 0.482 | **PPVT** |
|  | 0.629 | 0.386 | **CVLT 30s delay** |
|  | 0.576 | 0.169 | **CVLT recognition** |
|  | 0.486 | 0.212 | **Verbal fluency (sem.)** |
|  | 0.492 | 0.201 | **CVLT total recall** |
|  | 0.358 | 0.037 | **MMSE** |
|  | -0.080 | -0.489 | **Forward digit span** |
|  | -0.019 | -0.483 | **Backward digit span** |
|  | 0.335 | -0.094 | MTMT time |
|  | 0.266 | -0.136 | Verbal fluency (phon.) |
|  | 0.271 | -0.191 | Age |
|  | 0.241 | -0.194 | Sex |
|  | 0.204 | -0.200 | CDR language |
|  | 0.103 | -0.247 | Education |
|  | 0.086 | -0.276 | MTMT correct lines |
|  | 0.137 | -0.358 | CDR sum of boxes |
|  | 0.023 | -0.442 | CDR behavior |
| **LV-II** | 0.598 | 0.301 | **Backward digit span** |
|  | 0.510 | 0.197 | **Verbal fluency (phon.)** |
|  | 0.568 | 0.134 | **MMSE** |
|  | 0.496 | 0.131 | **Forward digit span** |
|  | -0.102 | -0.426 | **MTMT time** |
|  | -0.127 | -0.491 | **CDR sum of boxes** |
|  | -0.124 | -0.578 | **Age** |
|  | -0.097 | -0.614 | **BNT** |
|  | 0.384 | -0.038 | CVLT 30s delay |
|  | 0.354 | -0.053 | CVLT total recall |
|  | 0.334 | -0.044 | MTMT correct lines |
|  | 0.302 | -0.050 | Verbal fluency (sem.) |
|  | 0.424 | -0.059 | CVLT 10m delay |
|  | 0.315 | -0.162 | CVLT recognition |
|  | 0.284 | -0.142 | Sex |
|  | 0.302 | -0.203 | CDR language |
|  | 0.126 | -0.312 | Education |
|  | 0.083 | -0.314 | CDR behavior |
|  | -0.007 | -0.475 | PPVT |
| **LV-III** | 0.599 | 0.285 | **CDR behavior** |
|  | -0.032 | -0.372 | **CDR language** |
|  | -0.335 | -0.695 | **Age** |
|  | 0.402 | -0.055 | CDR sum of boxes |
|  | 0.079 | -0.300 | Education |
|  | 0.012 | -0.443 | MTMT correct lines |
|  | 0.406 | -0.051 | CVLT recognition |
|  | 0.425 | -0.103 | Forward digit span |
|  | 0.390 | -0.103 | Verbal fluency (phon.) |
|  | 0.387 | -0.127 | MTMT time |
|  | 0.420 | -0.255 | CVLT 10m delay |
|  | 0.281 | -0.206 | CVLT total recall |
|  | 0.339 | -0.309 | CVLT 30s delay |
|  | 0.255 | -0.278 | MMSE |
|  | 0.226 | -0.259 | Sex |
|  | 0.330 | -0.370 | PPVT |
|  | 0.360 | -0.441 | BNT |
|  | 0.228 | -0.367 | Backward digit span |
|  | 0.170 | -0.314 | Verbal fluency (sem.) |
| **LV-IV** | 0.704 | 0.195 | **CDR sum of boxes** |
|  | 0.628 | 0.217 | **CDR behavior** |
|  | 0.513 | 0.002 | **Verbal fluency (phon.)** |
|  | 0.581 | -0.011 | Forward digit span |
|  | 0.080 | -0.318 | CDR language |
|  | 0.656 | -0.037 | Age |
|  | 0.546 | -0.132 | BNT |
|  | 0.435 | -0.202 | PPVT |
|  | 0.469 | -0.252 | Backward digit span |
|  | 0.317 | -0.201 | Sex |
|  | 0.311 | -0.226 | CVLT total recall |
|  | 0.259 | -0.241 | Verbal fluency (sem.) |
|  | 0.357 | -0.344 | CVLT 30s delay |
|  | 0.324 | -0.376 | MMSE |
|  | 0.176 | -0.253 | MTMT time |
|  | 0.166 | -0.299 | MTMT correct lines |
|  | 0.140 | -0.279 | Education |
|  | 0.073 | -0.450 | CVLT recognition |
|  | 0.173 | -0.623 | CVLT 10m delay |


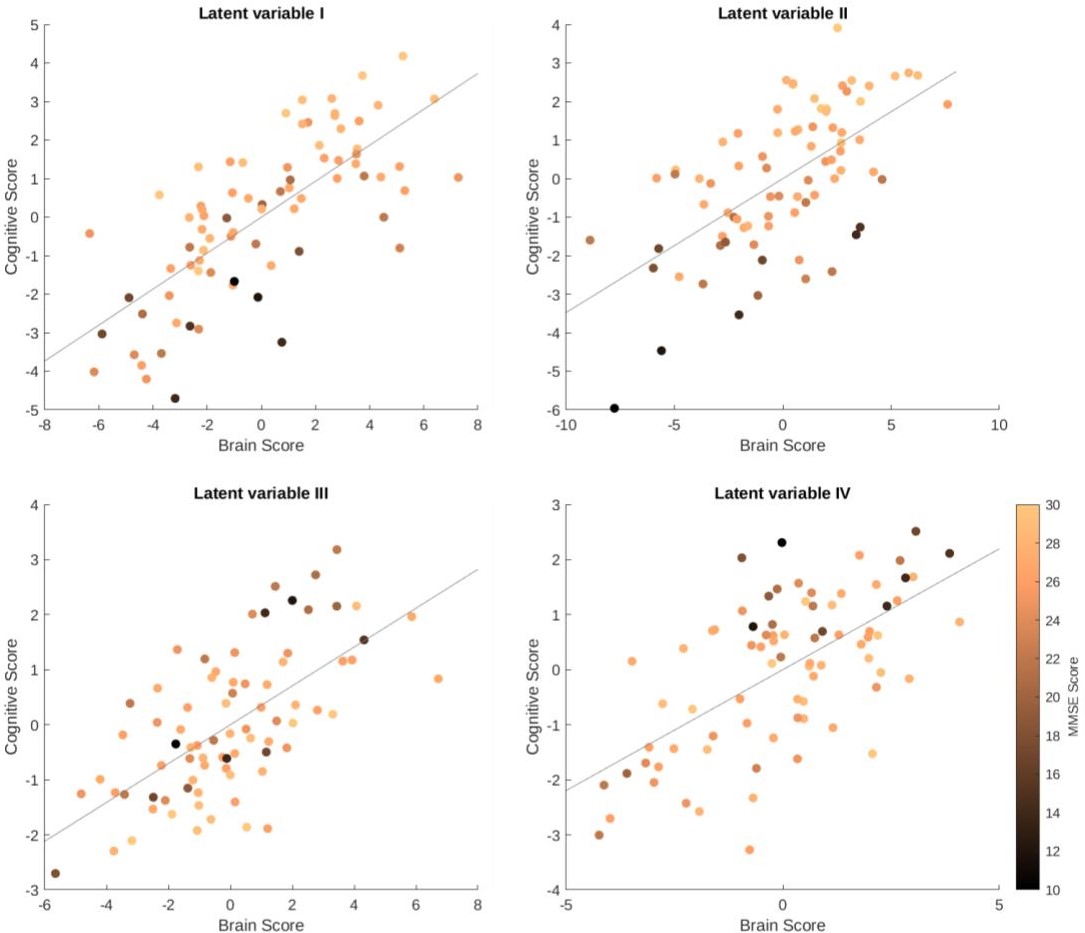


**Supplementary Figure 2.**  **Individual participants' brain versus cognitive PLS score for latent variables I to IV.** Individual brain and cognitive scores were calculated by projecting (multiplying) brain and cognitive profiles for each latent variable onto each patient’s data (N=78). Each datapoint represents one patient (N=78). MMSE scores for the respective patient are denoted in color (lighter colours show higher performance). The grey line is a least-squares line, indicating the association between brain and cognitive scores. This plot was used to visually (not statistically) assess the direction of the relationship between brain and cognitive scores in each latent variable, based on MMSE performance of the participants.


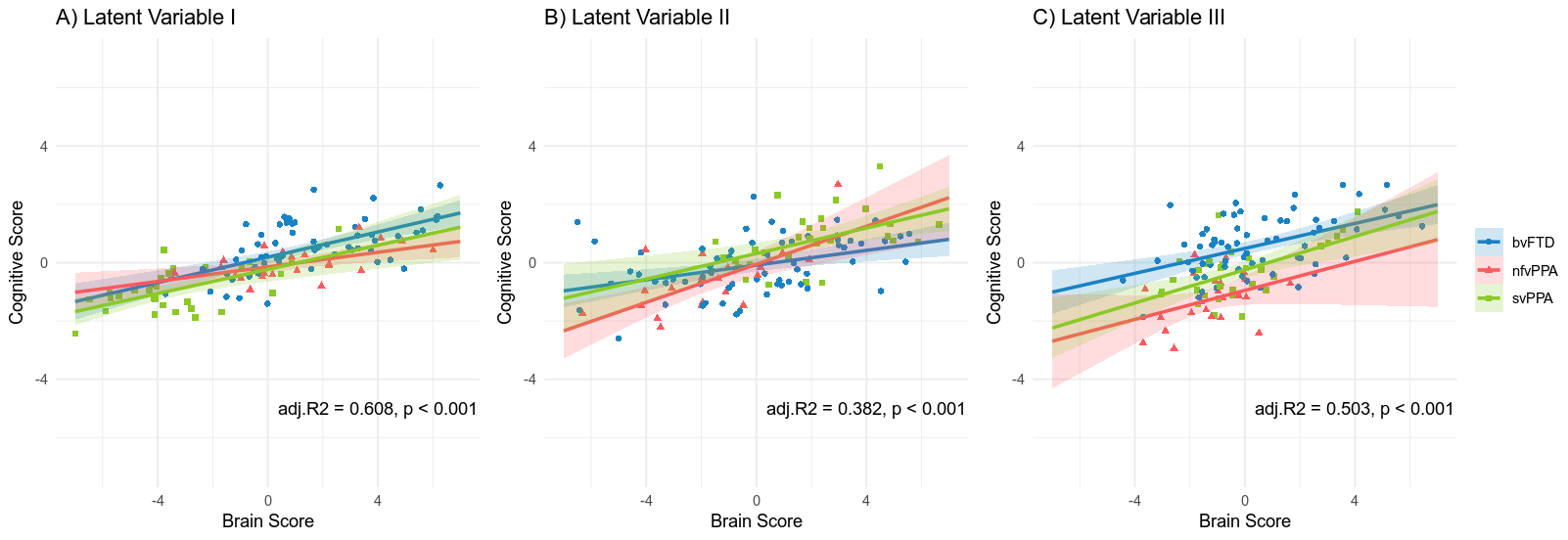
**
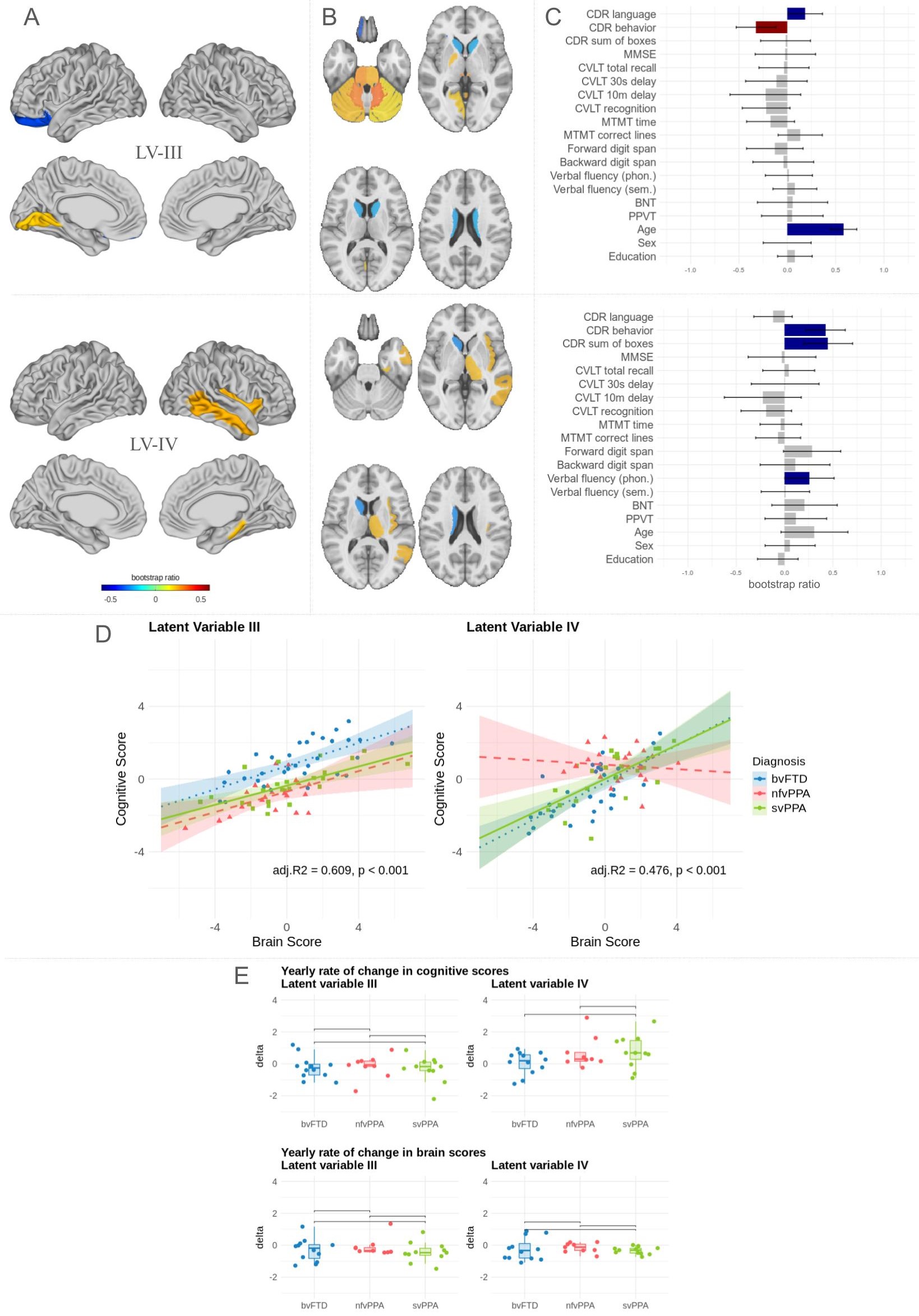
**

**Supplementary Figure 3. Latent variables III and IV (LVs III - IV) obtained from the PLS analysis.** PLS analysis in the maximal model including 78 FTD patients.

A, Brain pattern bootstrap ratios in MNI space, surface projection. Maps only include regions that significantly contribute to the LV, as estimated by bootstrap resampling of the 78 participants (p < 0.05) and confidence intervals. Cooler colours denote shrinkage/atrophy, warmer colour denote expansion.

B, Brain pattern bootstrap values in MNI space, horizontal view illustrating subcortical structures and cerebellum.

C, Pattern of demographic and cognitive test scores. The effect size estimates are derived from SVD analysis and the confidence intervals and significance levels are calculated by bootstrap resampling of the 78 participants (p < 0.05). Color blue (right) denotes lower scores in a test, red (left) denotes higher scores.

D, Individual patients’ brain versus cognitive PLS score and group differences between FTD variants for LV-III (adjusted R2 = 0.609, p < 0.001) and LV-IV (adjusted R2 = 0.476, p < 0.001). Individual brain and cognitive scores were calculated by projecting (multiplying) brain and cognitive profiles for each latent variable onto each patient’s data. We then applied linear regression models to compare the association of brain and cognitive scores between FTD subtypes. Each datapoint represents one patient (N=78). bvFTD = behavioral variant FTD (dotted line); svPPA = semantic variant primary progressive aphasia (solid line); nfvPPA = nonfluent variant primary progressive aphasia (dashed line).

E, Boxplots showing the longitudinal progression of brain and cognition scores for latent variables (LVs) III and IV in three FTD subtypes. Data points show the yearly rate of change in scores between the baseline and 1-year follow-up visit in FTD patients with longitudinal data, separated by FTD variant. Each datapoint represents one patient (N=32). Group differences were not significant based on unpaired t-tests with Tukey HSD correction for multiple comparisons comparing the FTD groups. bvFTD = behavioral variant FTD; svPPA = semantic variant PPA; nfvPPA = nonfluent variant PPA.

**Supplementary Table 8. Differences in the predictions of the PLS model for the different FTD subtypes in latent variables (LVs) III and IV, as determined by a linear regression model.** P-values are reported after correction for multiple comparisons using a false discovery rate controlling method with a significance threshold of 0.05. Significant intercept or slope differences are indicated by an asterisk. bvFTD = behavioral variant FTD; svPPA = semantic variant PPA; nfvPPA = nonfluent variant PPA.

| **Contrast** |  | **tStat** | **p-value** |
| --- | --- | --- | --- |
| **Latent variable III** | | | |
| bvFTD vs svPPA | Intercept | 4.848 | **<0.001*** |
|  | Slope | 0.628 | 0.532 |
| bvFTD vs nfvPPA | Intercept | 5.559 | **<0.001*** |
|  | Slope | 0.308 | 0.759 |
| svPPA vs nfvPPA | Intercept | 1.253 | 0.214 |
|  | Slope | -0.151 | 0.880 |
| **Latent variable IV** | | | |
| bvFTD vs svPPA | Intercept | -0.710 | 0.480 |
|  | Slope | 0.318 | 0.751 |
| bvFTD vs nfvPPA | Intercept | -3.108 | **0.003*** |
|  | Slope | 3.390 | **0.001*** |
| svPPA vs nfvPPA | Intercept | -2.424 | **0.018*** |
|  | Slope | 2.939 | **0.004*** |

**Description of LVs III and IV: PLS and linear regression**

The demographic and behavioral features contributing to the third LV (LV-III) were composed of higher age and CDR language subscore and lower CDR behavior subscores. The corresponding brain pattern consisted of reduced cerebellar white matter and vermal lobules I-V, and increased DBM values in the frontal lobe and right parahippocampal area.

The fourth LV (LV-IV) was characterized by higher impairment in the CDR sum of boxes and behavior subscale and phonological verbal fluency. The corresponding atrophy pattern was largely located in the right hemisphere (cerebellum, basal forebrain, thalamus, nucleus accumbens, superior frontal gyrus).

In LV-III, the bvFTD subtype had a significantly higher intercept compared to both the svPPA variant (tStat=-4.85, p<0.001) and the nfvPPA variant (tStat=-5.56, p<0.001). For LV-IV, the nfvPPA group had significantly higher intercepts than other groups (svPPA: tStat=2.42, p<0.02, bvFTD: tStat=3.12, p<0.003) as well as a different slope (svPPA: tStat=-2.94, p<0.05, bvFTD: tStat=-3.39, p<0.002), indicating LV-IV reflects the clinical/neural patterns of svPPA and bvFTD as opposed to nfvPPA.

**
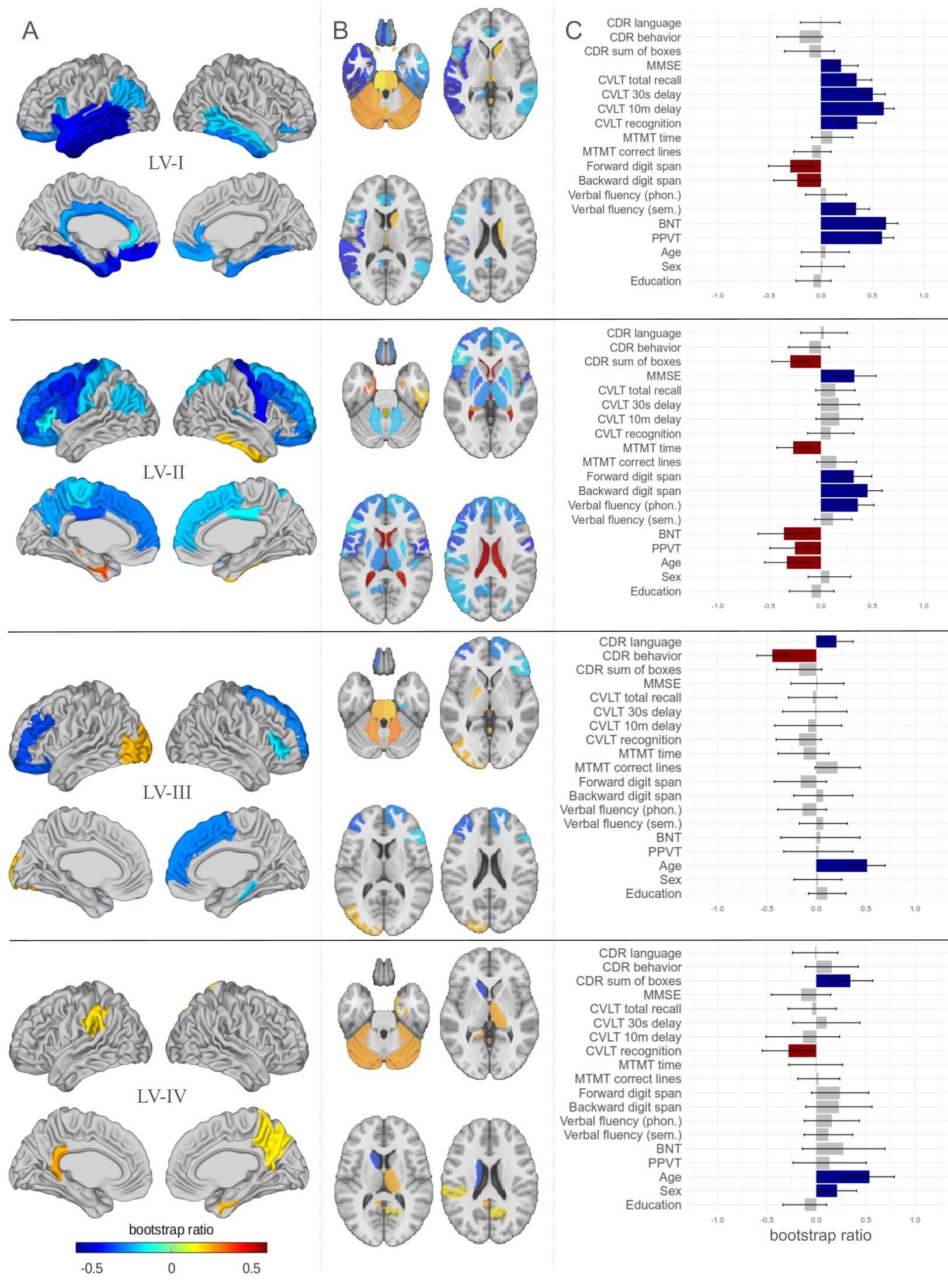
**

**Supplementary Figure 4. Latent variables I-IV (LVs I - IV) obtained from the PLS analysis after regression out the effects of healthy age, sex, and education (using w-scores).** PLS analysis in the maximal model including 78 FTD patients.

A, Brain pattern bootstrap ratios in MNI space, surface projection. Maps only include regions that significantly contribute to the LV, as estimated by bootstrap resampling of the 78 participants (p < 0.05) and confidence intervals. Cooler colours denote shrinkage/atrophy, warmer colour denote expansion.

B, Brain pattern bootstrap values in MNI space, horizontal view illustrating subcortical structures and cerebellum.

C, Pattern of demographic and cognitive test scores. The effect size estimates are derived from SVD analysis and the confidence intervals and significance levels are calculated by bootstrap resampling of the 78 participants (p < 0.05). Color blue (right) denotes lower scores in a test, red (left) denotes higher scores.

**
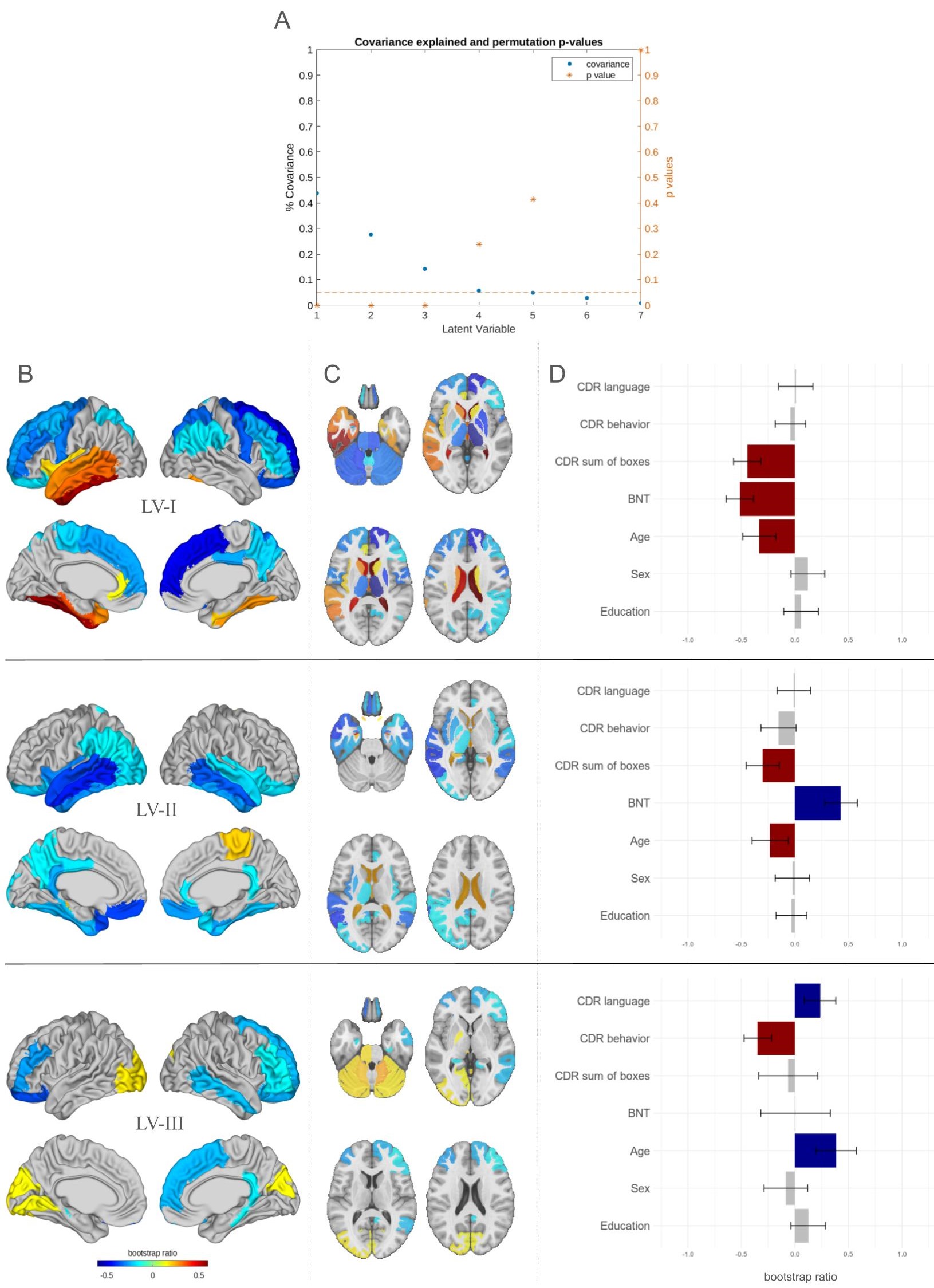
**

**Supplementary Figure 5. Latent variables I-IV (LVs I - IV) obtained from the PLS analysis with minimal clinical and demographic variables (CDR, BNT, age, sex, education),** including 120 FTD patients.

A, Covariance explained and permutation p-values for all latent variables. Blue circles show the amount of covariance between cognitive and atrophy data that is explained by each LV. Orange asterisks denote p-values associated with each LV, based on permutation tests. Latent variable I (LV-I, covariance explained = 43.81%, *p* < 0.001), latent variable II (LV-II, covariance explained 27.68%, *p* < 0.001), and latent variable III (LV-III, covariance explained =14.21%, *p* < 0.001), are described further based on p-value (permuted p < 0.05).

B, Brain pattern bootstrap ratios in MNI space, surface projection. Maps only include regions that significantly contribute to the LV, as estimated by bootstrap resampling of the 120 participants (p < 0.05) and confidence intervals. Cooler colours denote shrinkage/atrophy, warmer colour denote expansion.

C, Brain pattern bootstrap values in MNI space, horizontal view illustrating subcortical structures and cerebellum.

D, Pattern of demographic and cognitive test scores. The effect size estimates are derived from SVD analysis and the confidence intervals and significance levels are calculated by bootstrap resampling of the 120 participants (p < 0.05). Color blue (right) denotes lower scores in a test, red (left) denotes higher scores.

**Supplementary Table 9. Results of the classification analysis, specifically models run for validation (age-matched sample, CDR-matched sample, classification in longitudinal data, excluding demographic data from the model).** Machine learning was used to predict diagnosis of the FTD variant for each participant in a k-fold cross-validation. Results are presented as mean values and SD. bvFTD = behavioral variant FTD; svPPA = semantic variant PPA; nfvPPA = nonfluent variant PPA.

|  | **Classification accuracy** | **Sensitivity** | **Specificity** | **Balanced accuracy** |
| --- | --- | --- | --- | --- |
| **Maximal model (including 16 cognitive scores, age/sex/education)** | | | | |
| **Combination brain + cognition** | | | | |
| in CDR matched sample  brain + cognition | 88.88% (sd=1.51%) | bvFTD: 85.84% (sd=2.63%)  svPPA: 93.25% (sd=2.12%)  nfvPPA: 87.45% (sd=2.80%) | bvFTD: 92.89% (sd=1.64%)  svPPA: 96.78% (sd=1.16%)  nfvPPA: 93.69% (sd=0.77%) | bvFTD: 89.37%  svPPA: 95.02%  nfvPPA: 90.57% |
| age-matched sample  brain + cognition | 88.74% (sd=2.06%) | bvFTD: 73.79% (sd=4.31%)  svPPA: 99.38% (sd=1.88%)  nfvPPA: 91.19% (sd=4.08%) | bvFTD: 98.28% (sd=2.10%)  svPPA: 96.80% (sd=0.66%)  nfvPPA: 87.77% (sd=2.01%) | bvFTD: 86.03%  svPPA: 98.09%  nfvPPA: 89.48% |
| excluding demographics  brain + cognition | 87.51% (sd=1.28%) | bvFTD: 85.94% (sd=1.75%)  svPPA: 93.32% (sd=1.89%)  nfvPPA: 83.00% (sd=2.64%) | bvFTD: 90.78% (sd=1.61%)  svPPA: 96.00% (sd=0.62%)  nfvPPA: 94.07% (sd=1.14%) | bvFTD: 88.36%  svPPA: 94.66%  nfvPPA: 88.54% |
| **Cognition patterns only** | | | | |
| longitudinal data  cognition | 92.47% (sd=1.99%) | bvFTD: 92.00% (sd=1.64%)  svPPA: 86.82% (sd=5.69%)  nfvPPA: 100% (sd=0%) | bvFTD: 100% (sd=0%)  svPPA: 100% (sd=0%)  nfvPPA: 89.52% (sd=2.77%) | bvFTD: 96.00%  svPPA: 93.41%  nfvPPA: 94.76% |
| in CDR matched sample  cognition | 85.88% (sd=1.41%) | bvFTD: 84.20% (sd=3.08%) svPPA: 84.42% (sd=2.19%)  nfvPPA: 89.75% (sd=1.10%) | bvFTD: 93.66% (sd=1.30%)  svPPA: 93.40% (sd=0.38%)  nfvPPA: 91.88% (sd=1.64%) | bvFTD: 88.93%  svPPA: 88.91%  nfvPPA: 90.82% |
| age-matched sample  cognition | 86.09% (sd=2.18%) | bvFTD: 71.29% (sd=5.37%)  svPPA: 93.38% (sd=1.49%)  nfvPPA: 91.75% (sd=3.54%) | bvFTD: 98.75% (sd=1.54%)  svPPA: 94.20% (sd=2.35%)  nfvPPA: 85.80% (sd=1.68%) | bvFTD: 85.02%  svPPA: 93.79%  nfvPPA: 88.78% |
| excluding demographics  cognition | 86.27% (sd=1.44%) | bvFTD: 86.47% (sd=2.31%)  svPPA: 89.36% (sd=2.97%)  nfvPPA: 82.29% (sd=2.15%) | bvFTD: 92.41% (sd=1.59%)  svPPA: 94.40% (sd=0.32%)  nfvPPA: 92.54% (sd=1.66%) | bvFTD: 89.44%  svPPA: 91.88%  nfvPPA: 87.41% |
| **Brain patterns only** | | | | |
| longitudinal data  brain | 71.94% (sd=3.08%) | bvFTD: 67.92% (sd=6.08%)  svPPA: 89.27% (sd=3.51%)  nfvPPA: 56.11% (sd=6.19%) | bvFTD: 79.35% (sd=3.46%)  svPPA: 94.95% (sd=1.14%)  nfvPPA: 83.52% (sd=3.05%) | bvFTD: 73.64%  svPPA: 92.11%  nfvPPA: 69.81% |
| in CDR matched sample  brain | 68.99% (sd=2.78%) | bvFTD: 58.20% (sd=5.19%)  svPPA: 93.42% (sd=2.07%)  nfvPPA: 53.15% (sd=6.26%) | bvFTD: 77.39% (sd=2.97%)  svPPA: 93.40% (sd=1.91%)  nfvPPA: 82.69% (sd=2.18%) | bvFTD: 67.80%  svPPA: 93.41%  nfvPPA: 67.92% |
| age-matched sample  brain | 76.33% (sd=3.16%) | bvFTD: 61.36% (sd=4.44%)  svPPA: 99.94% (sd=0.63%)  nfvPPA: 65.81% (sd=7.61%) | bvFTD: 86.00% (sd=3.83%)  svPPA: 96.67% (sd=0%)  nfvPPA: 81.97% (sd=2.07%) | bvFTD: 73.68%  svPPA: 98.30%  nfvPPA: 73.89% |
| excluding demographics  brain | 67.92% (sd=2.25%) | bvFTD: 62.41% (sd=3.28%)  svPPA: 93.40% (sd=2.00%)  nfvPPA: 46.00% (sd=6.60%) | bvFTD: 73.93% (sd=3.14%)  svPPA: 92.75% (sd=0.70%)  nfvPPA: 83.88% (sd=1.74%) | bvFTD: 68.17%  svPPA: 93.08%  nfvPPA: 64.94% |
| **Minimal model (including CDR, BNT, age/sex/education)** | | | | |
| **Combination brain + cognition** | | | | |
| in CDR matched sample  brain + cognition | 81.54% (sd=1.42%) | bvFTD: 79.24% (sd=2.13%)  svPPA: 90.00% (sd=0.00%)  nfvPPA: 75.14% (sd=4.40%) | bvFTD: 87.54% (sd=1.86%)  svPPA: 91.75% (sd=0.98%)  nfvPPA: 92.03% (sd=1.09%) | bvFTD: 83.39%  svPPA: 90.88%  nfvPPA: 83.59% |
| age-matched sample  brain + cognition | 83.78% (sd=1.76%) | bvFTD: 73.24% (sd=3.68%)  svPPA: 94.12% (sd=0%)  nfvPPA: 84.00% (sd=3.84%) | bvFTD: 94.91% (sd=2.00%)  svPPA: 97.06% (sd=0%)  nfvPPA: 83.71% (sd=1.84%) | bvFTD: 84.07%  svPPA: 95.59%  nfvPPA: 83.85% |
| sample in site 1 (UCSF)  brain + cognition | 87.15% (sd=1.11%) | bvFTD: 88.37% (sd=2.06%)  svPPA: 87.07% (sd=1.09%)  nfvPPA: 84.83% (sd=2.35%) | bvFTD: 90.28% (sd=1.43%)  svPPA: 96.22% (sd=0.71%)  nfvPPA: 93.47% (sd=1.28%) | bvFTD: 89.33%  svPPA: 91.64%  nfvPPA: 89.15% |
| excluding demographics  brain + cognition | 81.66% (sd=1.32%) | bvFTD: 84.37% (sd=1.86%)  svPPA: 87.66% (sd=0.68%)  nfvPPA: 65.65% (sd=4.21%) | bvFTD: 82.07% (sd=1.88%)  svPPA: 92.36% (sd=0.78%)  nfvPPA: 94.40% (sd=0.96%) | bvFTD: 83.22%  svPPA: 90.01%  nfvPPA: 80.03% |
| **Cognition only** | | | | |
| longitudinal data  cognition | 84.95% (sd=1.22%) | bvFTD: 85.08% (sd=1.72%)  svPPA: 89.05% (sd=1.97%)  nfvPPA: 79.13% (sd=2.25%) | bvFTD: 91.40% (sd=1.18%)  svPPA: 90.36% (sd=0.91%)  nfvPPA: 95.03% (sd=1.28%) | bvFTD: 88.24%  svPPA: 89.71%  nfvPPA: 87.08% |
| in CDR matched sample  cognition | 73.98% (sd=1.42%) | bvFTD: 80.00% (sd=2.37%)  svPPA: 71.53% (sd=1.86%)  nfvPPA: 63.91% (sd=3.22%) | bvFTD: 81.73% (sd=1.84%)  svPPA: 87.86% (sd=1.02%)  nfvPPA: 89.67% (sd=1.48%) | bvFTD: 80.87%  svPPA: 79.70%  nfvPPA: 76.79% |
| age-matched sample  cognition | 77.63% (sd=2.28%) | bvFTD: 74.53% (sd=6.32%)  svPPA: 76.12% (sd=2.02%)  nfvPPA: 82.24% (sd=1.87%) | bvFTD: 94.50% (sd=1.49%)  svPPA: 88.79% (sd=1.95%)  nfvPPA: 83.15% (sd=2.83%) | bvFTD: 84.46%  svPPA: 82.46%  nfvPPA: 82.69% |
| sample in site 1 (UCSF)  cognition | 79.36% (sd=1.08%) | bvFTD: 86.20% (sd=1.56%)  svPPA: 72.90% (sd=2.05%)  nfvPPA: 74.13% (sd=1.89%) | bvFTD: 88.87% (sd=1.02%)  svPPA: 90.58% (sd=0.91%)  nfvPPA: 89.43% (sd=0.89%) | bvFTD: 87.53%  svPPA: 81.74%  nfvPPA: 81.78% |
| excluding demographics  cognition | 69.31% (sd=1.50%) | bvFTD: 90.43% (sd=1.78%)  svPPA: 33.44% (sd=3.21%)  nfvPPA: 63.59% (sd=3.71%) | bvFTD: 69.82% (sd=1.68%)  svPPA: 88.79% (sd=2.02%)  nfvPPA: 89.99% (sd=0.77%) | bvFTD: 80.13%  svPPA: 61.12%  nfvPPA: 76.79% |
| **CDR-only model (including CDR, age/sex/education)** | | | |  |
| CDR  Brain + cognition | 73.63% (sd=1.40%) | bvFTD: 80.46% (sd=1.53%)  svPPA: 64.78% (sd=2.73%)  nfvPPA: 68.38% (sd=3.80%) | bvFTD: 77.17% (sd=1.83%)  svPPA: 87.20% (sd=1.14%)  nfvPPA: 92.43% (sd=0.99%) | bvFTD: 78.82%  svPPA: 75.99%  nfvPPA: 80.40% |
| CDR  cognition | 63.87% (sd=1.70%) | bvFTD: 83.14% (sd=1.79%)  svPPA: 25.58% (sd=3.07%)  nfvPPA: 65.52% (sd=4.27%) | bvFTD: 64.48% (sd=1.53%)  svPPA: 83.52% (sd=1.93%)  nfvPPA: 91.26% (sd=0.81%) | bvFTD: 73.81%  svPPA: 54.55%  nfvPPA: 78.39% |
| **BNT-only model (including BNT, age/sex/education)** | | | |  |
| BNT  brain + cognition | 76.74% (sd=1.16%) | bvFTD: 83.72% (sd=1.37%)  svPPA: 87.84% (sd=1.32%)  nfvPPA: 43.04% (sd=3.40%) | bvFTD: 69.27% (sd=1.82%)  svPPA: 95.33% (sd=0.47%)  nfvPPA: 93.02% (sd=0.99%) | bvFTD: 76.50%  svPPA: 91.59%  nfvPPA: 68.03% |
| BNT  cognition | 71.77% (sd=0.99%) | bvFTD: 84.75% (sd=1.30%)  svPPA: 78.12% (sd=0.00%)  nfvPPA: 28.13% (sd=3.47%) | bvFTD: 65.41% (sd=1.64%)  svPPA: 89.97% (sd=0.37%)  nfvPPA: 93.96% (sd=1.04%) | bvFTD: 75.08%  svPPA: 84.05%  nfvPPA: 61.05% |


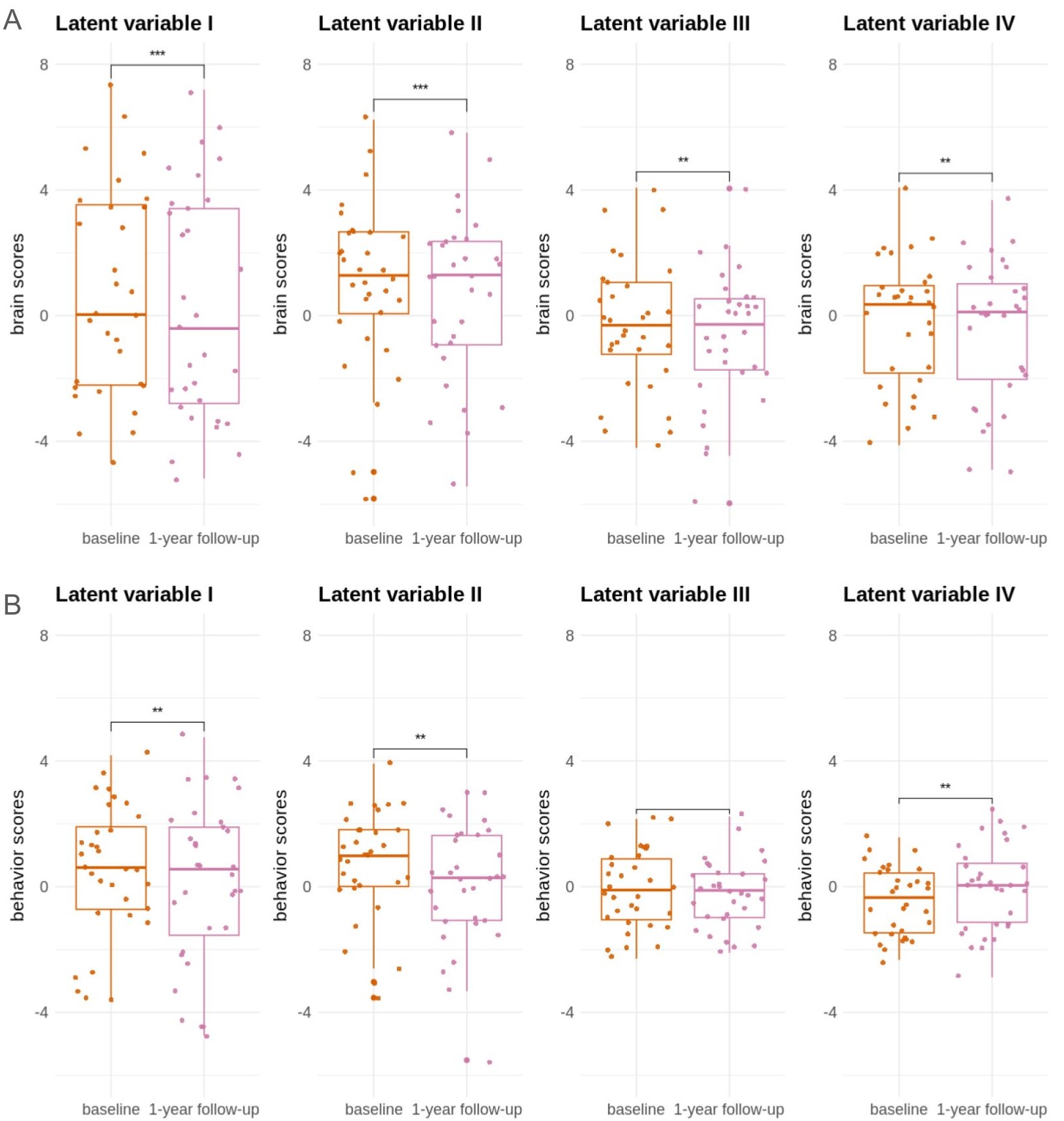


**Supplementary Figure 6. Boxplots showing the longitudinal progression of brain and cognition scores for latent variables (LVs) I - IV.** Progression of brain and behavior scores over one year follow-up in all participants with longitudinal data. Each datapoint represents one patient (N=32). Asterisks indicate significant group differences based on paired t-tests. A, Baseline and longitudinal brain scores (LV I: p=0.007, LV II: p<0.001, LV III: p<0.001, LV IV: p<0.003). B, Baseline and longitudinal cognition scores (I: *p*=0.006, II: *p*<0.002, IV: p<0.007).
